# In vivo single cell analysis reveals Gata2 dynamics in cells transitioning to hematopoietic fate

**DOI:** 10.1084/jem.20170807

**Published:** 2018-01-02

**Authors:** Christina Eich, Jochen Arlt, Chris S. Vink, Parham Solaimani Kartalaei, Polynikis Kaimakis, Samanta A. Mariani, Reinier van der Linden, Wiggert A. van Cappellen, Elaine Dzierzak

**Affiliations:** 1 Department of Cell Biology, Erasmus Stem Cell Institute, Erasmus Medical Center, Rotterdam, Netherlands; 2 Department of Pathology, Erasmus Optical Imaging Centre, Erasmus Medical Center, Rotterdam, Netherlands; 3 School of Physics and Astronomy, The University of Edinburgh, Edinburgh, Scotland, UK; 4 Centre for Inflammation Research, Queen's Medical Research Institute, The University of Edinburgh, Edinburgh, Scotland, UK

## Abstract

Cell fate is established through coordinated gene expression programs in individual cells. Regulatory networks that include the Gata2 transcription factor play central roles in hematopoietic fate establishment. Although Gata2 is essential to the embryonic development and function of hematopoietic stem cells that form the adult hierarchy, little is known about the in vivo expression dynamics of Gata2 in single cells. Here, we examine Gata2 expression in single aortic cells as they establish hematopoietic fate in *Gata2Venus* mouse embryos. Time-lapse imaging reveals rapid pulsatile level changes in Gata2 reporter expression in cells undergoing endothelial-to-hematopoietic transition. Moreover, Gata2 reporter pulsatile expression is dramatically altered in *Gata2^+/−^* aortic cells, which undergo fewer transitions and are reduced in hematopoietic potential. Our novel finding of dynamic pulsatile expression of *Gata2* suggests a highly unstable genetic state in single cells concomitant with their transition to hematopoietic fate. This reinforces the notion that threshold levels of Gata2 influence fate establishment and has implications for transcription factor–related hematologic dysfunctions.

## Abstract (precis)

Eich et al. reveal the dynamic expression of the Gata2 transcription factor in single aortic cells transitioning to hematopoietic fate by vital imaging of *Gata2Venus* mouse embryos. Pulsatile expression level changes highlight an unstable genetic state during hematopoietic cell generation.

## Introduction

During a short window of developmental time, hematopoietic stem cells (HSCs) arise from the transdifferentiation of specialized endothelial cells (ECs) lining the major embryonic vasculature. In the mouse, this endothelial-to-hematopoietic transition (EHT) occurs at embryonic day (E) 10.5 and is best characterized by the emergence of clusters of hematopoietic stem and progenitor cells (HSPCs) from the aortic endothelium of the aorta-gonad-mesonephros (AGM) region (Dzierzak and Medvinsky, 2008; Dzierzak and Speck, 2008). The transition involves changes in the transcriptional program of a subset of (hemogenic) ECs to a program promoting HSPC identity. RNA-sequencing data from our group and others has shown that expression of a group of “heptad” transcription factors (TFs; Wilson et al., 2010; Lichtinger et al., 2012; Solaimani Kartalaei et al., 2015; Goode et al., 2016) increases during EHT (Solaimani Kartalaei et al., 2015), suggesting that heptad TFs could act as a transcriptional hub for the regulation of EHT.

Gata2, one of the heptad TFs, is crucial for the generation of HSCs. *Gata2* is expressed in the mouse embryo in the primitive streak, some ECs of the paired and midgestation dorsal aorta, and vitelline/umbilical arteries (Minegishi et al., 1999; Robert-Moreno et al., 2005; Kaimakis et al., 2016). At the time of definitive HSPC formation and during EHT, it is expressed in hemogenic ECs (HECs) and intra-aortic hematopoietic cluster cells (IAHCs). *Gata2^−/−^* embryos suffer from fetal liver anemia and die in midgestation at the time of HSC generation (Ng et al., 1994; Tsai et al., 1994; Orlic et al., 1995; Tsai and Orkin, 1997; Minegishi et al., 1999; Nardelli et al., 1999; Ling et al., 2004; Robert-Moreno et al., 2005; Khandekar et al., 2007; de Pater et al., 2013). *Gata2* heterozygous mutant (*Gata2^+/−^*) embryos are severely affected in the production of early progenitors (Tsai et al., 1994) and have greatly reduced numbers of HECs, IAHCs, HPCs, and HSCs (Ling et al., 2004; Khandekar et al., 2007; de Pater et al., 2013; Gao et al., 2013). *Gata2^+/−^* HSCs are qualitatively defective (Ling et al., 2004; Rodrigues et al., 2005). Thus, Gata2 has distinct roles during the different stages of hematopoietic development and is a pivotal regulator of EHT cell transition, HSC generation, and function (de Pater et al., 2013). How Gata2 controls these different processes and how levels of Gata2 expression influence cell fate decisions remain elusive.

Recent studies have identified a growing list of TFs that show pulsatile dynamic behavior (Lahav et al., 2004; Nelson et al., 2004; Cai et al., 2008; Cohen-Saidon et al., 2009; Locke et al., 2011; Levine et al., 2013; Purvis and Lahav, 2013; Ryu et al., 2016; Zambrano et al., 2016). A pulse is detected when a critical threshold of TF molecules accumulate and ends when they are degraded/deactivated. The presence of pulsatile expression for various regulators in bacteria (Locke et al., 2011; Young et al., 2013), yeast (Garmendia-Torres et al., 2007; Dalal et al., 2014), and the mammalian stress response and signaling pathways (Lahav et al., 2004; Nelson et al., 2004; Kageyama et al., 2008; Cohen-Saidon et al., 2009; Kholodenko et al., 2010; Tay et al., 2010; Batchelor et al., 2011; Albeck et al., 2013; Yissachar et al., 2013) suggests that it is a common process. Pulsing may provide a time-based mode of regulation, where an input typically modulates the pulse frequency, amplitude, and/or duration of individual TFs to control downstream target gene expression. This dynamic behavior and pulsatile expression of TFs in single cells is implicated in cell transitions and fate decisions (Nelson et al., 2004; Shimojo et al., 2008; Kobayashi et al., 2009; Tay et al., 2010; Pourquié, 2011; Imayoshi et al., 2013; Kueh et al., 2013, 2016; Neuert et al., 2013; Stern and Piatkowska, 2015) and includes, for example the NF-κb and Notch signaling pathways (Kim et al., 2013; Levine et al., 2013; Purvis and Lahav, 2013; Isomura and Kageyama, 2014).

Although much information is emerging on transcriptomic signatures and molecules affecting the development of the hematopoietic system (Lichtinger et al., 2012; Swiers et al., 2013; Solaimani Kartalaei et al., 2015; Goode et al., 2016; Zhou et al., 2016), dynamic expression is still a largely unexplored area. We set out to examine the dynamics of *Gata2* expression during the establishment of hematopoietic fate in the aortic endothelium, because *Gata2* is a downstream target of the Notch pathway (Robert-Moreno et al., 2005; Gama-Norton et al., 2015) and is known to affect EHT (Kumano et al., 2003; Ling et al., 2004; de Pater et al., 2013), and the dosage of *Gata2* is of major importance for normal hematopoietic development (Ling et al., 2004; Khandekar et al., 2007; Tipping et al., 2009; de Pater et al., 2013; Gao et al., 2013). Here, we demonstrate for the first time the pulsatile expression of a *Gata2* reporter during the process whereby hematopoietic cells are generated from HECs. By vital imaging of single cells in the mouse embryonic aorta (WT and *Gata2* heterozygous mutant), we show that cell states during EHT correlate with *Gata2* reporter expression duration, levels (amplitude changes), and pulse periodicity, thus supporting the notion that Gata2 levels and dynamic behavior are linked to hematopoietic fate.

## Results

### Gata2 reporter expression in single cells is dynamic

**Figure 1.**
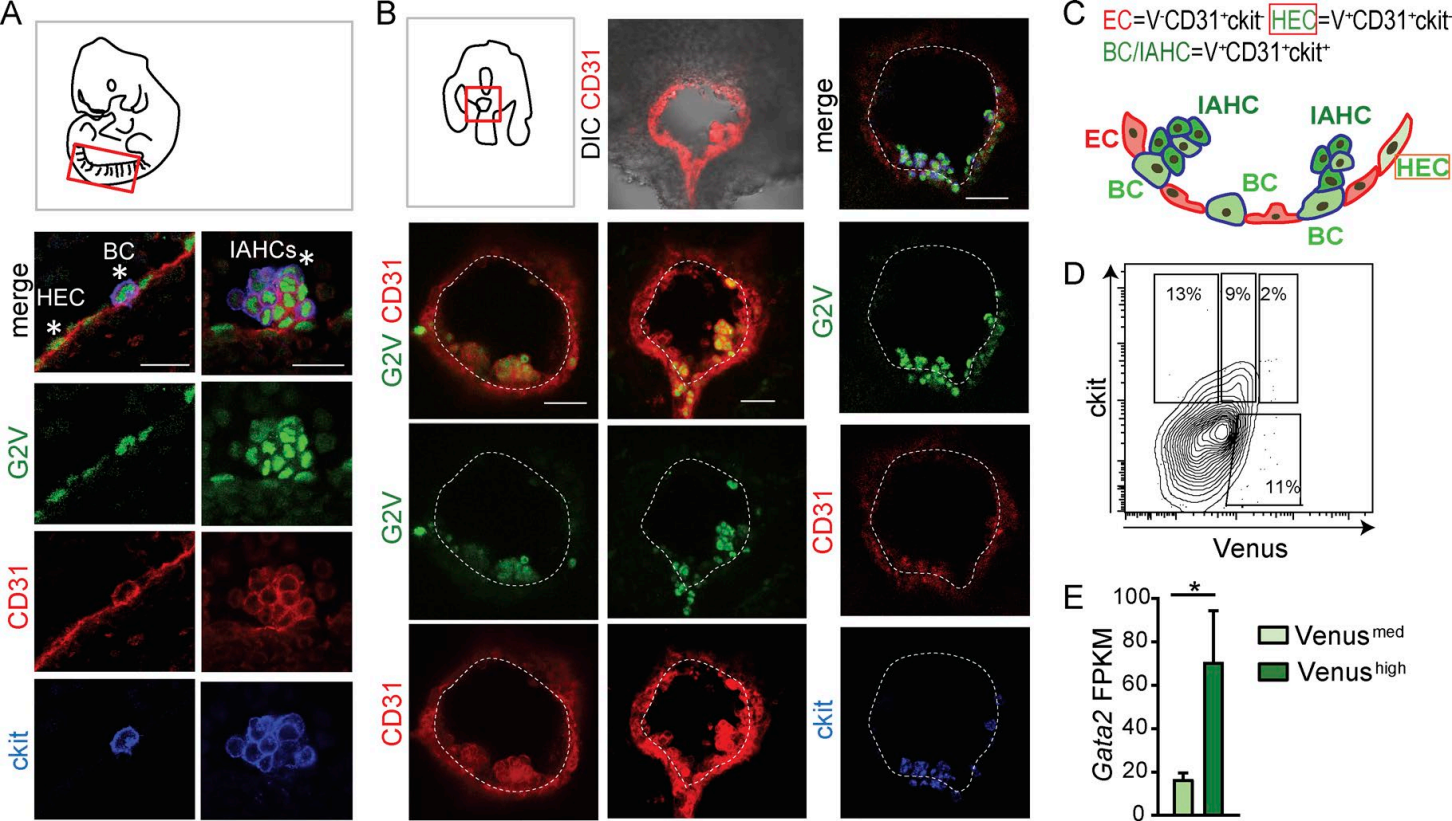
***G2V* reporter reveals expression of Venus in different EHT cell subsets. (A)** Diagram of a whole mount of a 35-SP embryo with the dorsal aorta outlined in red and confocal images of the ventral aspect of the *G2V* dorsal aorta. HECs and BCs (left) and IAHCs (right) are indicated by asterisks (CD31, red; ckit, blue; and Venus, green). Bars, 20 µm. **(B)** Diagram of an embryo (33–34 SPs) transversal slice prepared for vital confocal time-lapse imaging. Confocal images of representative immunostained *G2V* embryo traversal sections (CD31, red; ckit, blue; and Venus, green). Bars, 40 µm. Ventral side downward. DIC, differential interference contrast. **(C)** Schematic representation of Gata2 (green) expression in the different EHT cell subsets in the E10.5 mouse dorsal aorta (ventral aspect; ECs, red, red outline; HECs, light green, red outline; BCs, medium green, blue outline; and IAHCs, dark green, blue outline). Cells with blue outline express ckit in addition to CD31. **(D)** Flow cytometric contour plot of CD31 gated cells. Percentages of CD31^+^ckit^+^Venus^med^ and CD31^+^ckit^+^Venus^high^ expressing cells from E10.5 *G2V* AGMs. **(E)** Bar graph of *Gata2* transcription in E10.5 AGM *G2V* sorted CD31^+^ckit^+^Venus^med^ (light green) and CD31^+^ckit^+^Venus^high^ (dark green) cells. y axis shows FPKM values obtained from RNA-sequencing analysis. The data represent the mean ± SEM of three independent experiments and were compared using a Student’s *t* test (*, P = 0.0431).

Cell populations undergoing EHT in the mouse embryonic aorta are characterized by their localization, morphology, and expression of pivotal markers and TFs, including Gata2 (Kaimakis et al., 2016). To specifically examine Gata2 expression in single cells undergoing EHT, confocal imaging was performed on immunostained E10.5 AGM from *Gata2Venus* (*G2V*) reporter embryos (Kaimakis et al., 2016) in fixed whole-mount (Fig. 1 A) and vital transverse-section preparations (Fig. 1 B). Venus fluorescence reports *Gata2* expression (*IRES-Venus* insertion in *Gata2 3*′ *UTR*) without disrupting normal *Gata2* transcription, protein stability or function, or hematopoietic development (Kaimakis et al., 2016). Importantly, Gata2 is a short-lived protein (30–60 min; Minegishi et al., 2005; Lurie et al., 2008). Rather than using the GFP reporter (half-life = 9 h), the Venus reporter was chosen for this study because its half-life (60–120 min) closely reflects real-time Gata2 expression dynamics (Li et al., 1998). Vital imaging was performed on 150-µm transversal sections of E10.5 *G2V* embryos preinjected with antibodies against CD31 (endothelial and IAHC marker) and ckit (IAHC marker; Boisset et al., 2010; Yokomizo et al., 2012; Solaimani Kartalaei et al., 2015).

Venus expression was found in single cells of the aortic endothelium, cells bulging from the endothelial wall, and IAHCs (Fig. 1, A and B), all of which are CD31^+^. ECs (CD31^+^ckit^−^) are flattened ckit^−^ cells in the vascular wall, and for this study, G2V-expressing (V^+^) ECs are referred to as HECs. G2V-expressing ckit^+^ cells undergoing a change in morphology as they emerge from the wall are referred to as bulging cells (BCs; V^+^CD31^+^ckit^+^). IAHCs (V^+^CD31^+^ckit^+^) are the rounded cells found in clusters adjacent to the endothelial layer (Fig. 1 C). Flow cytometric analysis (FACS) showed that varying levels (medium and high) of Venus expression could be detected in the CD31^+^ckit^+^ cells (Fig. 1 D). Upon sorting CD31^+^ckit^+^ Venus^med^ and Venus^high^ cells, RNA-sequencing analysis (Fig. 1 E) showed medium and high levels of *Gata2* transcripts, respectively. Furthermore, Venus^med^ and Venus^high^ expression levels correctly reflect Gata2 protein levels, as confirmed by Western blotting of sorted cell fractions from adult *G2V* bone marrow (Fig. S1 A). Equivalent ratios of quantified Gata2 to Venus protein signal were found for all the sorted cell populations. Hence, the G2V reporter allows the accurate tracking of *Gata2* expression in single live cells during EHT.

**Figure 2.**
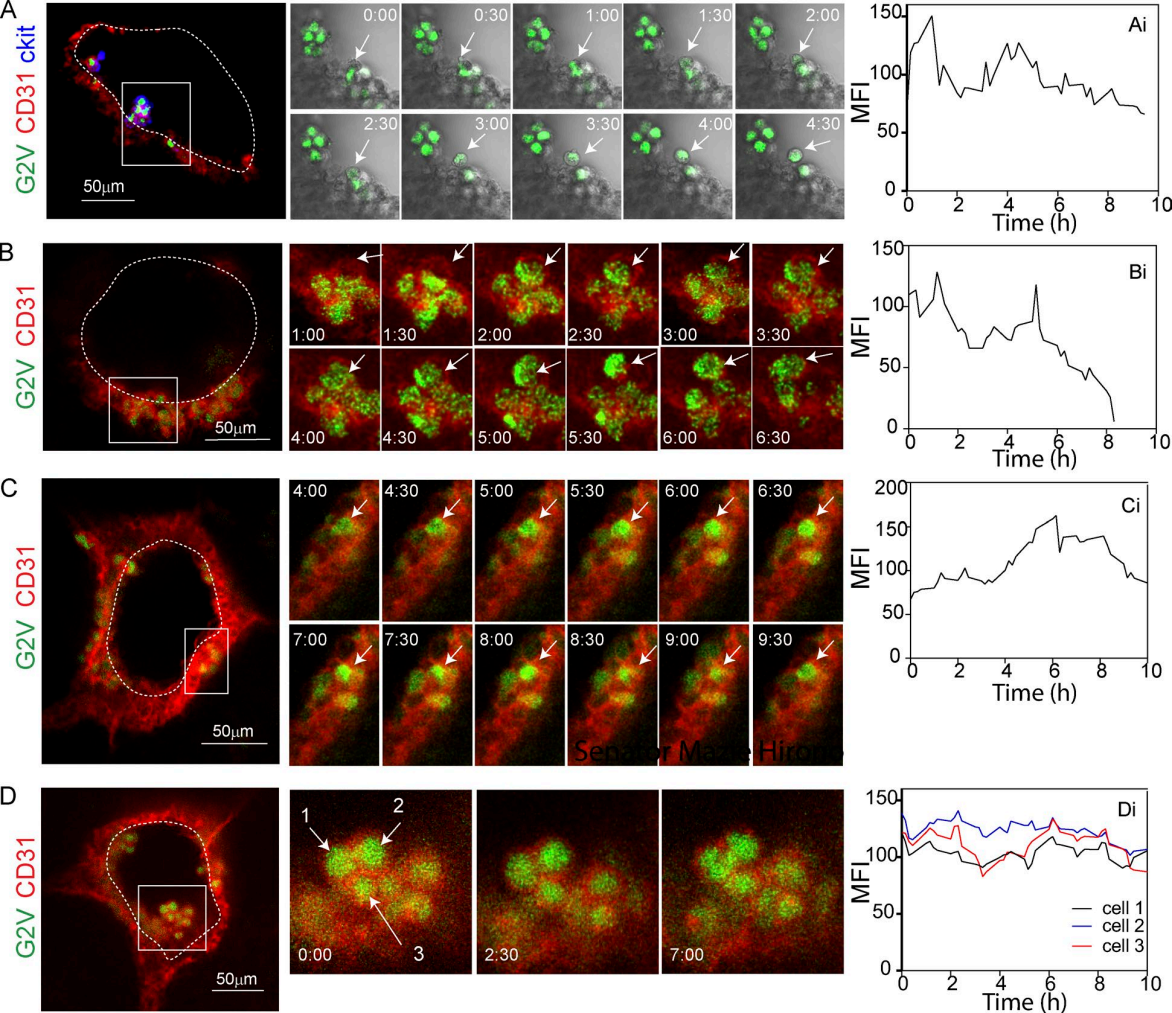
**Dynamic expression of the Gata2 reporter during EHT. (A–C)** Confocal time-lapse imaging of E10 (33–34 SPs) *G2V* embryos (Venus, green) immunostained with anti–CD31 (red) and anti–ckit (blue) antibodies (A) or anti–CD31 (red) antibody only (B and C). Arrows indicate cells undergoing EHT events. **(D)** Confocal time-lapse imaging of IAHC at E10 (33–34 SPs; Venus, green), immunostained with anti–CD31 (red) antibody. Arrows indicate three IAHC cells. **(Ai–Di)** Quantification of Venus MFI over time (hours) corresponding to the highlighted cells during the process of EHT (Ai–Ci) and in IAHC (Di). Transverse aortic sections were imaged for 10 h at 15-min intervals. A–C (middle) show 30-min snapshots, and D shows snapshots at 0, 2.5, and 7 h. Bars, 50 µm.

Vital imaging of *G2V* embryo transversal sections through the AGM was performed at 15-min intervals for 10–15 h (Videos 1, 2, and 3). Imaging data were examined for EHT events in which Venus-expressing (V^+^) hematopoietic cells emerge from the aortic wall. In 15 independent time-lapse imaging experiments with a total of 49 sections, we observed 13 EHT events of V^+^CD31^+^(ckit^+^) hematopoietic cells emerging from the V^+^CD31^+^(ckit^−^) endothelium of E10 embryos (32–37 somite pairs [SPs]; Fig. 2 and Videos 1, 2, and 3). Taking into account the thickness of the embryo section and length of the aorta (forelimb to hindlimb where IAHC are found), we calculated that there are ∼20 EHT events per embryo. This is in contrast to the 1.7 EHT events per embryo previously observed in the *Ly6aGFP* reporter aorta imaging studies (Boisset et al., 2010). In the cases in which we imaged an EHT event, visual analysis of time-lapse images revealed changes in the mean fluorescent intensity (MFI) suggestive of flexible and pulsatile Gata2 expression in BCs (Fig. 2, A–C) and, to a lesser extent, IAHCs (Fig. 2 D and Fig. S3 F).

**Figure 3.**
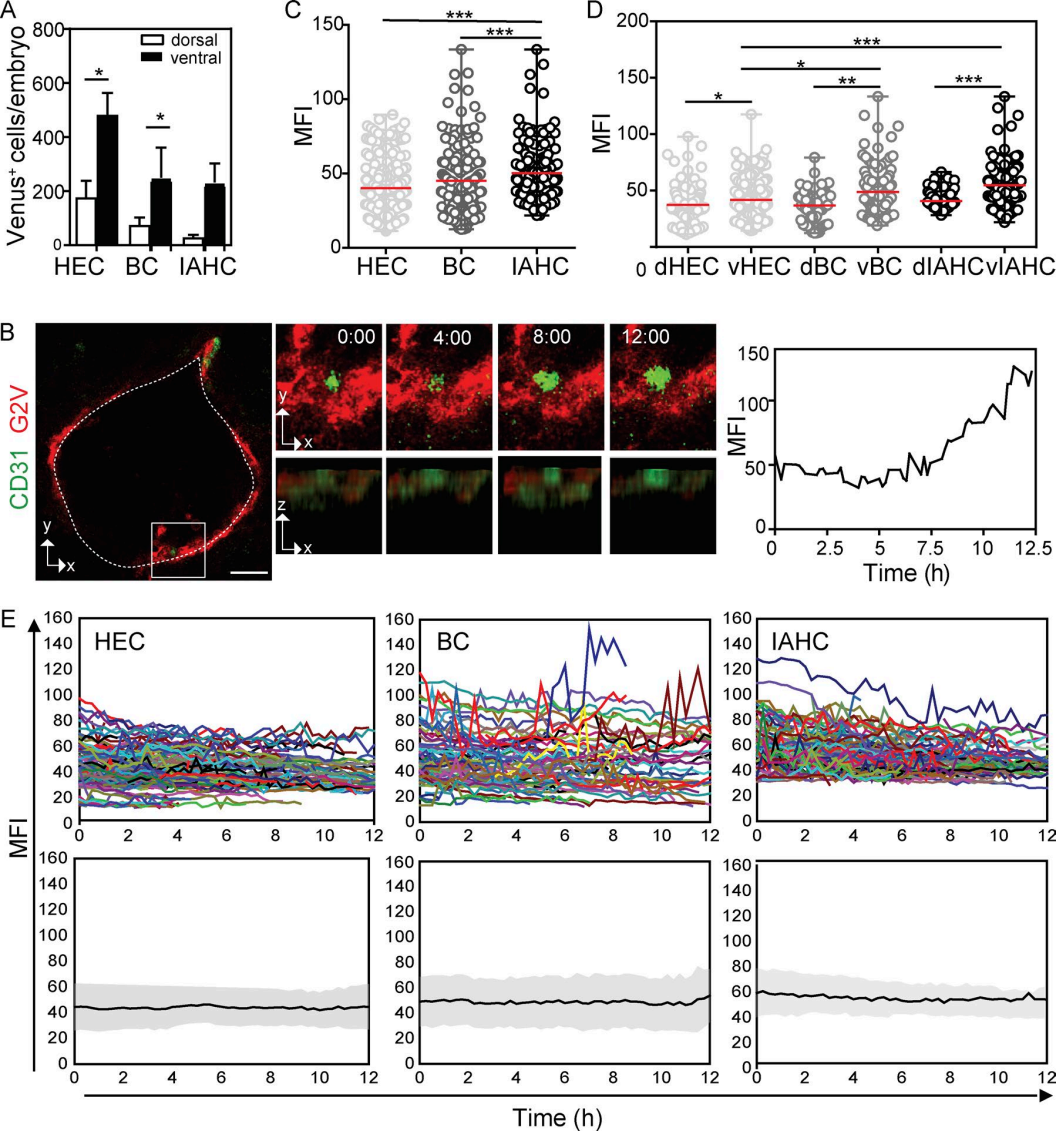
**Time-lapse imaging reveals differences in Gata2 dynamics between ECs, BCs, and IAHCs. (A)** Mean number of Venus^+^ EHT subset cells per *G2V* embryo according to their ventral and dorsal aortic location, as determined by microscopy. Venus^+^ HECs, BCs, or IAHCs were counted in the first frame of time-lapse imaging experiments (*n* = 15, 42 sections) of *G2V* embryo slices of 150 µm thickness and calculated per embryo (E10, 32–37 SPs). The data represent the mean ± SEM of 15 independent experiments, and dorsal and ventral location were compared using two-way ANOVA with Bonferroni post test (*, P < 0.05). **(B)** Left: Example of a BC showing decreasing and increasing levels of Venus expression during a 12-h imaging period, imaged at a time interval of 15 min. Middle: Three-dimensional projections (x-y axes and x-z axes) of the same BC as shown in the top panel with time (hours) indicated. Right: Corresponding Venus (green) MFI profile in time. Bar, 25 µm. Sections were stained with anti–CD31 (red) antibody. **(C)** Venus MFI (averaged over frames 3–12) in single EHT subset cells (HECs, BCs, and IAHCs; *n* = 15, 718 cells). The data represent the mean ± range. Statistical significance was calculated on the pooled data of 15 independent experiments using Mann–Whitney *U* test (***, P < 0.0001). **(D)** Venus MFI in single EHT subset cells plotted according to their dorsal (d) and ventral (v) location (*n* = 15, 718 cells). The data represent the mean ± range. Statistical significance was calculated on the pooled data of 15 independent experiments using Mann-Whitney *U* test (*, P < 0.0288; **, P = 0.0020; ***, P < 0.0001). **(E)** Top: Temporal variation of Venus MFI for individual Venus^+^ cells (colored lines) plotted according to their affiliation to one of the EHT subsets (EC, BC, or IAHC). Bottom: Gray bands represent the standard deviation of the mean MFI of all tracks (black line) over time.

V^+^ cells were counted in the first frame image of time-lapse experiments (*n* = 15; 1,126 cells). When the numbers were calculated per total aorta, 660 ± 87 V^+^ cells were found in the endothelial layer (HECs), followed by 305 ±131 in BCs (7% of which undergo EHT) and 266 ±132 in IAHCs. Highly sensitive FACS of E10.5 *G2V* AGMs confirmed the microscopy results, showing the highest numbers of V^+^ cells in the aortic endothelium (CD31^+^ckit^−^; 1,076 HECs) and fewer V^+^ cells in the CD31^+^ckit^+^ hematopoietic population (680 BCs and IAHCs; Table 1). These numbers are higher than what has been published previously for the *Ly6aGFP* reporter in the E10.5 AGM (831 HECs, and 261 BCs and IAHCs at 34 SPs; Solaimani Kartalaei et al., 2015), indicating that G2V expression is encompassing more EHT cells than the *Ly6aGFP* reporter (Fig. S1 B). Additionally, the majority of V^+^ HECs, BCs, and IAHCs are found on the ventral side of the aorta, with only 23% of V^+^ cells on the dorsal side (Fig. 3 A).

**Table 1.** Absolute cell numbers in each EHT phenotypic subset in the AGM as determined by flow cytometry

*Gata2* genotype	Stage (sp)	CD31^+^	CD31^+^ ckit^+^V^−^	CD31^+^ ckit^−^V^+^	CD31^+^ ckit^+^V^med^	CD31^+^ ckit^+^V^hi^	Number of experiments (embryos)
*+/+*	E10 (28–36)	10,860 ± 1,704	1,257 ± 264	1,076 ± 153	616 ± 223	64 ± 16	*n* = 5 (25)
+*/−*	E10 (30–36)	11,664 ± 1,992	1,049 ± 258	780 ± 179	186 ± 60	32 ± 6	*n* = 3 (19)

### Expression dynamics differ among HECs, BCs, and IAHCs

Time-lapse imaging allowed us to follow Venus expression up to 15 h without confounding bleaching effects. To quantitate Gata2 expression levels and dynamics during the transition of HECs to BCs and BCs to IAHCs, confocal images of single aortic cells were deconvoluted to improve the signal to noise ratio and analyzed using commercial and custom-made tools for tracking cells in four dimensions (Fig. S2). Only cells tracked for at least 10 consecutive frames were included, and tracking in three dimensions guaranteed that the observed changes in Venus intensity were not due to cells moving in or out of the imaging plane (Fig. 3 B). Venus MFI values plotted over time (Fig. S2; details in Materials and methods) showed expression in individual cells to be dynamic (Videos 1, 2, and 3), and as shown in Fig. 2, EHT was accompanied by increased, decreased, and/or alternating levels of Venus expression. Changes in Venus MFI were observed in both the raw and deconvoluted data, ruling out the possibility that deconvolution introduced artifacts (Fig. S3, A and B).

To examine whether expression levels in EHT subsets differed, MFI values of individual Venus^+^ cells in each subset were averaged for 10 consecutive time points (49 sections analyzed, 718 cells tracked). MFI values were significantly higher in IAHCs (50.3 ± 1.2) than in HECs (40.2 ±0.9; ***, P < 0.0001) and BCs (45.1 ± 1.7, **, P < 0.001; Fig. 3 C). MFI plots of EHT subsets according to their ventral or dorsal location revealed significantly higher levels of Venus expression in ventral HECs (*, P = 0.023), ventral BCs (**, P = 0.0036), and ventral IAHCs (***, P = 0.0001) than in dorsal ECs, dorsal BCs, and dorsal IAHCs, respectively (Fig. 3 D). Moreover, Venus MFI was significantly higher in ventral BCs (*, P = 0.0288) and ventral IAHCs (***, P < 0.0001) than in ventral HECs, suggesting that EHT cell identity is related to the level of *Gata2* expression.

To ensure that the differences in Venus MFI were not caused by noise inherent to the microscopy procedure, we performed time-lapse imaging of aorta sections that were labeled with DRAQ5, a fluorescent DNA-binding dye that should not undergo intensity changes during imaging. DRAQ5 was titrated to reach similar MFIs as Venus (Fig. S3 C). No dramatic intensity changes in DRAQ5 MFI were observed (Fig. S3 D). Visual inspection of the Venus intensity profiles of single HECs, BCs, and IAHCs over time (Fig. 3 E, top; and Fig. S3 F) revealed heterogeneity and differences in expression dynamics that are not observed in population analyses of these EHT subsets (Fig. 3 E, bottom).

The single cell analyses of Venus MFI showed higher amplitude and pulsatile changes in BCs and IAHCs than in HECs, indicating activated but unstable Gata2 expression as cells transit to hematopoietic fate. Because it was previously reported that Gata2 levels decrease during mitosis (Koga et al., 2007), we monitored our time-lapse videos for proliferation events. Although cell division was observed in 14% of IAHCs (Fig. S4), very few BCs (0.3%) and 0% of HECs divided (*n* = 15). As expected, Venus expression in IAHCs decreased during cell division. Because 3.5-fold more IAHCs (50%) showed fluctuating Venus expression than underwent cell division, and because no or very few proliferation events were detected in HECs and BCs during the imaging period, it is unlikely that cell division is responsible for the pulsatile behavior of Gata2 expression that we observe during the HEC to BC transition. To control for this, our results on Gata2 dynamic expression exclude cells undergoing mitosis.

### Gata2 reporter pulse amplitude and periodicity distinguishes EHT subsets

**Figure 4.**
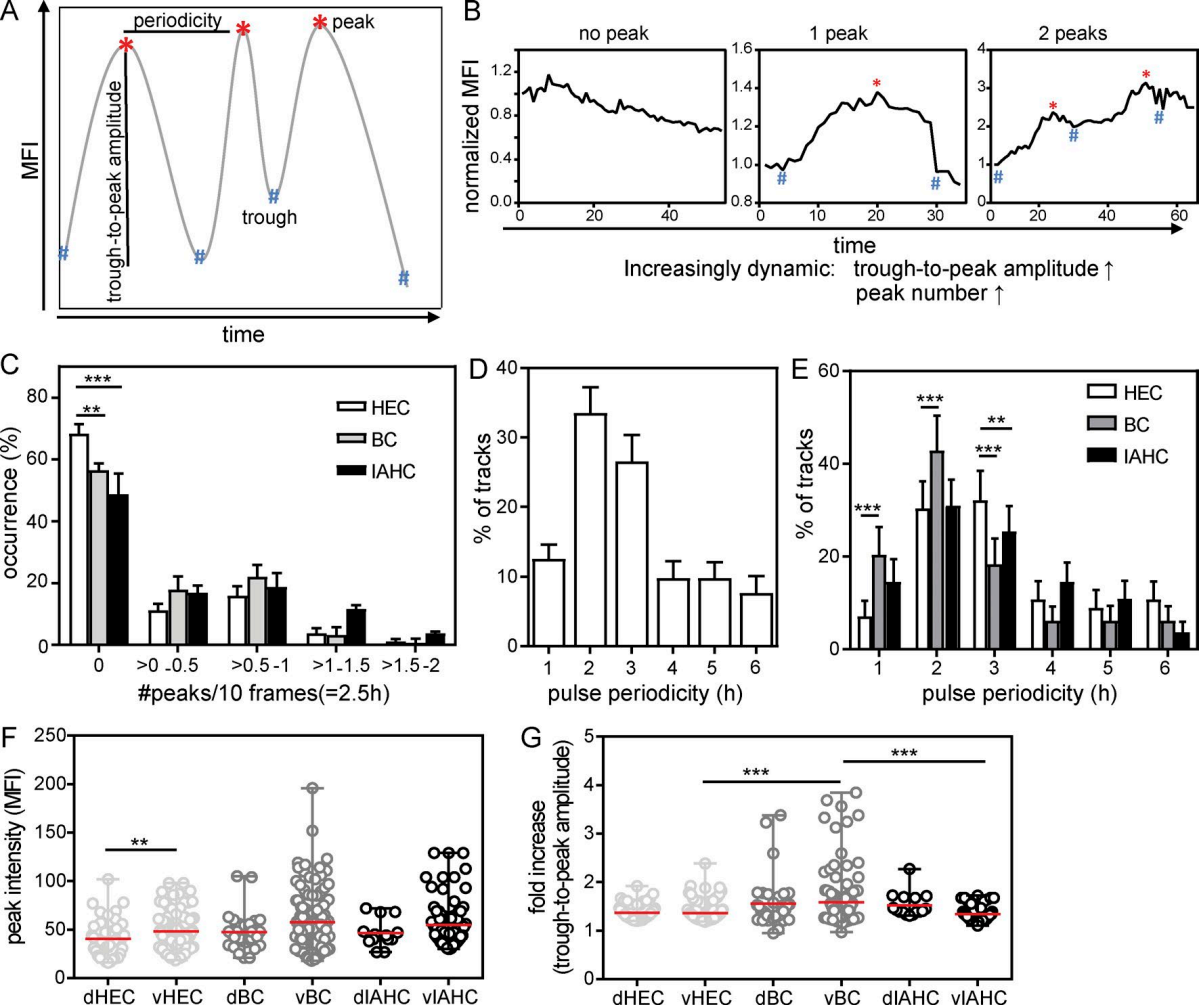
**Pulse frequency and amplitude of Venus expression distinguishes EHT subsets. (A)** Schematic representation of the automatic peak detection code. A local MFI maximum is considered a peak if it has at least a 15% higher intensity than its neighboring minima (see Materials and methods). The pulse period is the time between two adjacent peaks and the trough-to-peak amplitude the change between peak (highest value) and the preceding trough (lowest value). **(B)** Examples of normalized MFI profiles with no peak, one peak, and two pulse peaks showing increasing trough-to-peak amplitudes. **(C)** Distribution of the occurrence (percentage) of normalized pulse peak numbers in ECs, BCs, and IAHCs tracked over at least 10 consecutive frames (718 cells). To normalize for differences in track length, the data are presented as peaks per 10 frames (2.5 h) and represent the mean ± SEM (*n* = 15). Statistical significance was calculated using two-way ANOVA with Bonferroni post test (**, P < 0.01; ***, P < 0.001). **(D)** Distribution of the pulse periodicities of Venus^+^ cells showing at least two pulse peaks (*n* = 15, 221 cells). **(E)** Distribution of the pulse periodicities in EHT subset cells showing at least two pulse peaks (*n* = 15, 86 HECs, 80 BCs, and 55 IAHCs). The data represent the mean ± SD. Statistical significance was calculated on the pooled data (*n* = 15) using two-way ANOVA with Bonferroni post test (**, P ≤ 0.01; ***, P < 0.001). **(F and G)** Peak intensity (F) and trough-to-peak amplitude (G) in the EHT cell subsets, plotted according to their ventral (v) or dorsal (d) location in the aorta (*n* = 13, cells showing at least one peak: 170 HECs, 151 BCs, and 65 IAHCs). The data represent the mean ± range. Statistical significance was calculated on the pooled data (*n* = 15) using Mann–Whitney *U* test (**, P = 0.0054; ***, P < 0.0008).

Pulsatile behavior of regulatory molecules that relay information relevant to biological systems are characterized by their amplitude and periodicity of expression and/or activation state (Pourquié, 2011; Purvis and Lahav, 2013; Isomura and Kageyama, 2014). Expression amplitude is the maximal (peak) value a regulatory molecule attains during the observation period, whereas oscillation periodicity indicates the time between two adjacent peaks (Fig. 4 A). To obtain the quantitative changes (trough-to-peak amplitude) in Venus expression, the fold change between the MFI at the peak and at the preceding trough was calculated. We developed an automated data-processing methodology to quantify amplitude and pulse periodicity in individually tracked cells (for details, see Materials and methods).

To discriminate noise from real Venus peaks, we set a threshold in which the peak Venus MFI differs from its neighboring minima by ≥15% of the mean intensity of the track. We could detect zero, one, two, and three peaks in Venus MFI profiles for individual EHT cells tracked over at least 10 consecutive time frames (Fig. 4 B). The majority of cells showed constant Venus MFIs (0 peaks, 57%) through the imaging period, and two peaks were found for 21% of cells (Fig. S3 E). To account for the difference in track lengths of the individual cells imaged, we normalized the pulse data and calculated the occurrence of peaks per 2.5 h (10 frames). Within the EHT subsets (Fig. 4 C), a significantly higher percentage of HECs showed no peaks (68%) compared with BCs (56%: *, P < 0.05) and IAHCs (49%: ***, P < 0.001). Thus, Gata2 reporter expression showed a greater pulsatile behavior in BCs and IAHCs than in HECs (Fig. 4 C).

The pulse periodicity was calculated for all Venus^+^ cells (Fig. 4 D) and individual EHT subset cells (Fig. 4 E) showing at least two peaks. Approximately 55% of Venus^+^ cells showed pulse periodicities of 2–3 h (Fig. 4 D). EHT subset cells showed differing Venus periodicities (Fig. 4 E). As compared with HECs, more BCs showed periodicities of 1 h (20.4% vs. 7.1%: ***, P < 0.001) and 2 h (42.9% vs. 30.3%: ***, P < 0.001). Fewer BCs and IAHCs showed 3-h pulse periodicities (18.4%: ***, P < 0.001; and 25.5%: **, P < 0.01, respectively) as compared with HECs (32.1%). The majority of HECs (62%) and IAHCs (56%) showed peak Venus expression periodicities of 2 to 3 h, whereas the majority of BCs (63%) showed shorter pulse periodicities of 1 to 2 h, thus indicating a high rate of dynamic expression specifically in BC after emergence from the endothelium.

Because *Gata2* dosage is known to be important for the normal production of IAHCs and functional HSPCs, the maximal fluorescent protein abundance reached within an expression pulse was also calculated. In individual cells, the mean Venus peak MFI was higher in BCs (53 ± 2.3) and IAHCs (51 ± 2.3) than in HECs (46 ± 1.5). Ventrally localized cells showed higher Venus peak MFIs than dorsal cells (Fig. 4 F). Interestingly, and in contrast to the peak MFIs, trough-to-peak amplitude measurements (Fig. 4 G) showed significantly higher fold changes in expression in BCs than in HECs (***, P = 0.0008) and IAHCs (***, P = 0.0008). 12.8% of ventral BCs and 1.5% of dorsal BCs showed a trough-to-peak amplitude higher than twofold, in contrast to 0.5% of ventral HECs and 0% dorsal HECs. In IAHCs (ventral and dorsal), only 6% of cells showed trough-to-peak amplitudes higher than twofold. Thus, the degree of trough-to-peak amplitude changes in Venus expression in BCs suggest that the upstream and downstream signals will be variable in this EHT subset and could provide an explanation for the known phenotypic/functional heterogeneity of hematopoietic cells.

### Gata2 reporter levels, pulsatile behavior, and hematopoietic functions are interrelated

**Figure 5.**
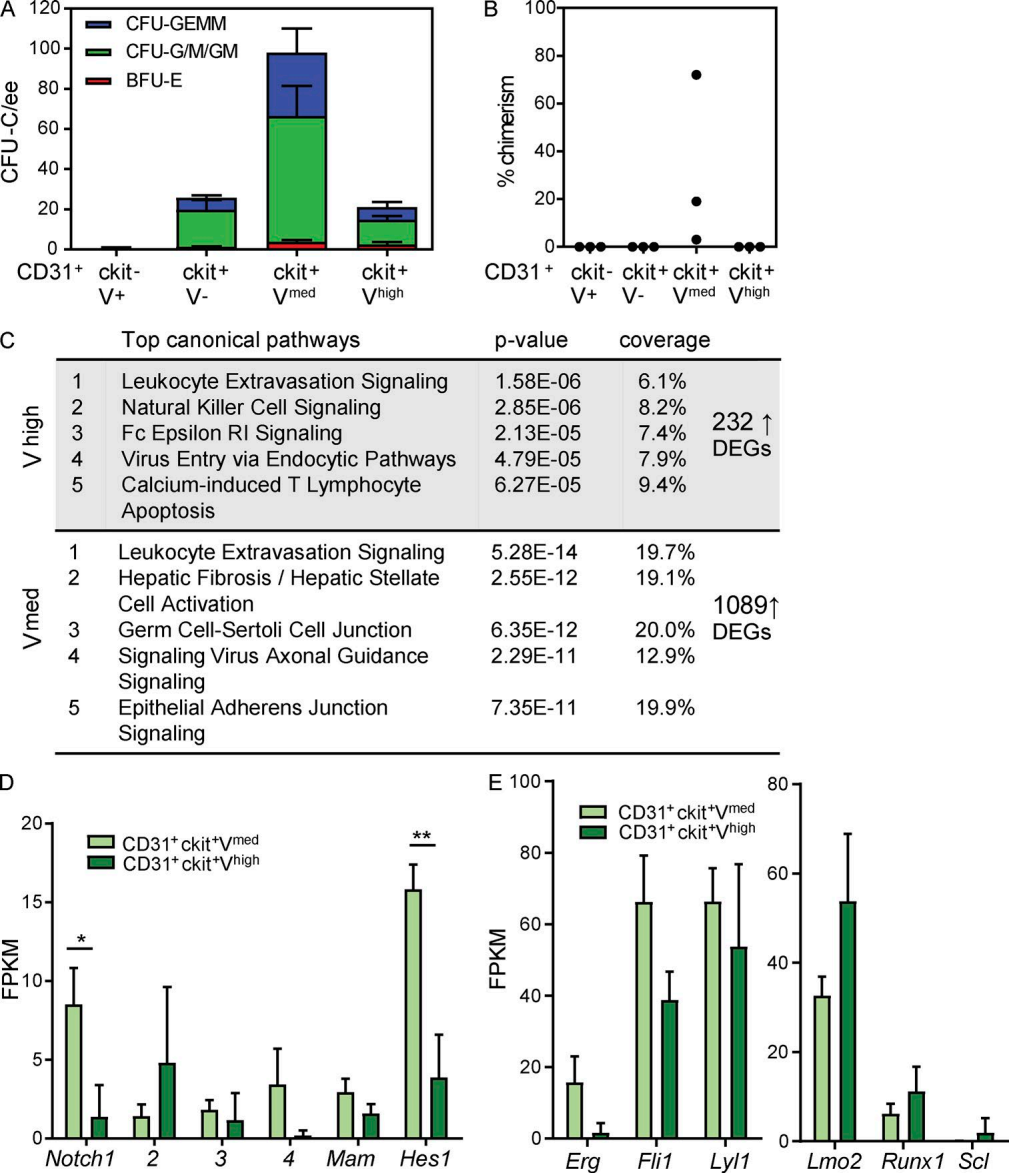
**Hematopoietic potential correlates with Venus expression levels. (A)** Hematopoietic progenitor numbers in E10.5 (30–36 SPs) CD31^+^ckit^+^V^med^ and CD31^+^ckit^+^V^high^ AGM sorted cells. Colony-forming unit-culture per embryo equivalent (CFU-C/ee) is shown, with colony types designated by colored bars. BFU-E, burst-forming unit erythroid; CFU-G/M/GM, CFU granulocyte, CFU macrophage, and CFU granulocyte-macrophage; CFU-GEMM, CFU granulocyte, erythroid, macrophage, megakaryocyte. The data represent the mean ± SEM of four independent experiments. **(B)** Percentage donor cell chimerism in recipient mice injected with CD31^+^ckit^−^V^+^, CD31^+^ckit^+^V^−^, CD31^+^ckit^+^V^med^, or CD31^+^ckitV^high^ sorted E11 (41–49 SPs) AGM cells. Engraftment at 4 mo after transplantation was determined by flow cytometric analysis of Ly5.1/Ly5.2 marker expression of peripheral blood cells. Each dot represents one recipient receiving 1.3 to 4.1 embryo equivalent (ee) of sorted AGM cells. The data represent the mean ± SD. *, P ≤ 0.024; **, P = 0.0085; ***, P = 0.0003). **(C)** Overrepresentation of up-regulated differentially expressed genes (DEGs) in E10.5 CD31^+^ckit^+^V^med^ and CD31^+^ckitV^high^ sorted cells in IPA canonical pathways. **(D and E)** Mean FPKM values for genes in the Notch pathway (D) and heptad factor genes in E10.5 CD31^+^ckit^+^V^med^ and CD31^+^ckitV^high^ sorted E10.5 AGM cells (E). The data were compared using Student’s *t* test (*, P = 0.0404; **, P = 0.0096). The data represent the mean ± SEM of three independent experiments.

Recently, we showed that most AGM HPCs and all HSCs are Gata2 expressing (Kaimakis et al., 2016). By FACS, CD31 and ckit expression discriminates ECs (CD31^+^ckit^−^V^−^), HECs (CD31^+^ckit^−^V^+^), and BCs/IAHCs (CD31^+^ckit^+^V^+^; Fig. 1 D). In line with our imaging data, CD31^+^ckit^+^V^+^ cells can be further divided into CD31^+^ckit^+^V^med^ and CD31^+^ckit^+^V^high^ fractions and assessed for hematopoietic function (Fig. 5, A and B). HPCs in E10 *G2V* AGMs were found to be highly enriched (71%) in the CD31^+^ckit^+^V^med^ fraction, with 14% in the CD31^+^ckit^+^V^high^ fraction (Fig. 5 A). Importantly, multipotent progenitors were highly enriched in the CD31^+^ckit^+^V^med^ fraction, and in vivo transplantations revealed that only this fraction contained HSCs (Fig. 5 B). Taken together, it is likely that the V^med^ cell fraction contains both BCs and IAHCs with multipotent hematopoietic activity.

Molecular characterization of these cell fractions (RNA sequencing) revealed differential expression of 1,321 genes, of which 1,089 genes showed down-regulated expression and 232 genes up-regulated expression in the CD31^+^ckit^+^V^high^ fraction. The Ingenuity Pathway Analysis tool revealed that the 232 up-regulated differentially expressed genes in the CD31^+^ckit^+^V^high^ fraction were significantly overrepresented in canonical pathways expressed by mature myeloid cell types (innate immune; Fig. 5 C). In contrast, the CD31^+^ckit^+^V^med^ cells showed enrichment for genes involved in leukocyte extravasation and epithelial adherence junction pathways. These data support the functional data to indicate that CD31^+^ckit^+^V^high^ cells are more differentiated hematopoietic cells and the CD31^+^ckit^+^V^med^ cells are immature progenitors and stem cells. Considering that the Notch signaling pathway is involved in hematopoietic cell development and IAHC formation (Kumano et al., 2003; Guiu et al., 2013) and that *Gata2* is a direct Notch target (Robert-Moreno et al., 2005), pathway-component analysis was performed. Significantly higher expression of *Notch1* and its target gene, *Hes1*, was found in the CD31^+^ckit^+^V^med^ fraction as compared with the CD31^+^ckit^+^V^high^ fraction (Fig. 5 D), supporting a role for Notch in V^med^ BCs and/or IAHCs. As expected, the heptad hematopoietic TF genes were expressed in both fractions (Fig. 5 E); expression of *Erg*, *Fli1*, and *Lyl1* was lower in CD31^+^ckit^+^V^high^ cells, and expression of *Lmo2*, *Runx1*, and *Scl* was higher in CD31^+^ckit^+^V^high^ cells. These data indicate a degree of molecular heterogeneity within V^+^ emerging hematopoietic cells.

**Figure 6.**
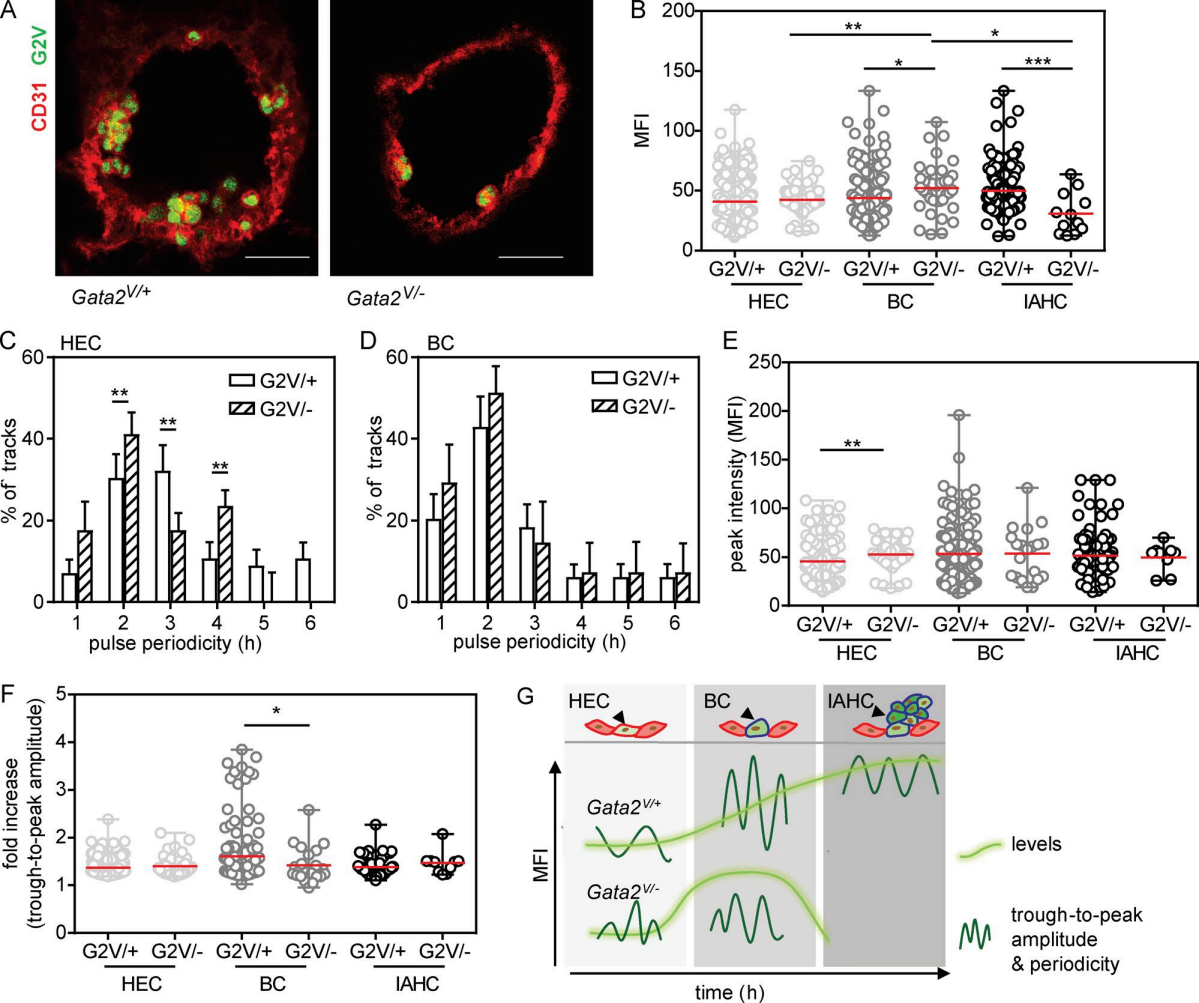
**Gata2 expression parameters, hematopoietic fate, and EHT are interrelated. (A)** Maximum projections of confocal time-lapse images of E10.5 *Gata2^V/+^* and *Gata2^V/−^* aortas immunostained with anti-CD31 (red; G2V, green). Bars, 40 µm. *Gata2^V/+^* and *Gata2^V/−^* embryos were harvested from the same litter. Ventral side downward. **(B)** Venus MFI (averaged over frames 3–12) in single *Gata2^V/-^* EHT subset cells (*n* = 6; 75 ECs, 37 BCs, and 12 IAHCs). The data were compared with *Gata2^V/+^* EHT subset cells using one-way ANOVA with Bonferroni post test (mean ± SD; *, P ≤ 0.024; **, P = 0.0085; ***, P = 0.0003). **(C and D)** Distribution of the pulse periodicities in Venus^+^ EHT subset cells: HECs (C) and BCs (D) from E10.5 *Gata2^V/−^* aortas showing at least two pulsatile peaks (*n* = 6; 18 HECs and 15 BCs). The data represent the mean ± SD. The data were compared with *Gata2^V/+^* HECs and BCs using two-way ANOVA with Bonferroni post test (**, P < 0.01). **(E and F)** Peak intensity (E) and trough-to-peak amplitude (F) in the *Gata2^V/−^* EHT subsets (*n* = 6, cells showing at least one peak: 36 ECs, 20 BCs, and 6 IAHCs). The data represent the mean ± range. The data were compared with Gata2^V/+^ EHT subset cells using Mann–Whitney *U* test (*, P = 0.0321; **, P = 0.0056). **(G)** Model of Gata2 expression dynamics and pulsatile characteristic during EHT. EHT cell types (top) are shown with accompanying Gata2 dynamic expression changes EHT directly below. G2V MFI (bright green) and pulse parameters (dark green sinusoids) are shown for *Gata2^V/+^* (middle) and *Gata2^V/−^* (bottom) EHT subset cells.

To further examine whether Gata2 pulse periodicity, trough-to-peak amplitude, and hematopoietic functions in the aorta are related, we crossed *G2V* (*G2^V/V^*) and *Gata2^+/−^* mice (Tsai et al., 1994; C-terminal zinc-finger deletion) to obtain embryos (*Gata2^V/−^*) with one mutated and one functional allele of *Gata2* (Fig. S5 A). It is known that *Gata2* heterozygous mutant embryos have a greatly reduced number of IAHCs, HPCs, and HSCs (Tsai and Orkin, 1997; Ling et al., 2004; Khandekar et al., 2007; de Pater et al., 2013; Gao et al., 2013), and HSCs are qualitatively defective (Ling et al., 2004; Rodrigues et al., 2005). As found by vital imaging (*n* = 6, 18 sections), the number of V^+^ IAHCs and BCs was lower in *Gata2^V/−^* aortas than in *Gata2^V/+^* aortas, whereas the number of V^+^ HECs was similar between *Gata2^V/−^* and *Gata2^V/+^* aortas (Fig. 6 A; compare Fig. S5 B with Fig. 3 A). Venus^+^ BC and IAHC were almost exclusively on the ventral side. In line with our microscopy data, FACS analysis of *Gata2^V/−^* aortas showed reduced numbers of CD31^+^ckit^+^V^+^ cells (Table 1). To examine Gata2 protein levels, we sorted E10.5 *Gata2^V/+^* and *Gata2^V/−^* AGM cells into V^+^ and V^−^ fractions and performed Western blotting (Fig. S5, C and D). Equal levels of Gata2 protein were found in *Gata2^V/+^* and *Gata2^V/−^* AGMs. Upon examination of the MFI values of all imaged V^+^ cells in *Gata2^V/−^* aortas (Fig. 6 B), no differences in MFI were detected between *Gata2^V/+^* and *Gata2^V/−^* HECs. However, in contrast to *Gata2^V/+^* aortas, where Venus expression was highest in IAHCs, Venus expression in *Gata2^V/−^* aortas was highest in BCs. In the few remaining *Gata2^V/−^* IAHCs, Venus expression was lower than in *Gata2^V/+^* IAHCs.

Further analysis of Gata2 reporter pulsatile expression parameters showed no difference in peak number distribution between *Gata2^V/+^* and *Gata2^V/−^* HECs; in both cases, 30% of HECs showed pulsatile expression (Fig. S5 E). A trend toward reduced numbers of BCs with pulsatile expression was found in *Gata2^V/−^* embryos (31%) as compared with *Gata2^V/+^* embryos (46%; Fig. S5 F). Despite no peak number differences in HECs, 51% of *Gata2^V/−^* HECs showed pulse periodicities of ≤2 h as compared with 25% in *Gata2^V/+^* HECs (Fig. 6 C), indicating that bursts of Gata2 expression are more frequent in *Gata2^V/−^* HECs. A similar trend toward reduced pulse periodicities was also found in *Gata2^V/−^* BCs as compared with *Gata2^V/+^* BCs (Fig. 6 D). Because IAHC were highly reduced in *Gata2^V/−^* aortas, we could not image sufficient numbers of IAHCs with pulsatile characteristics to reliably calculate periodicities. Within a pulse, *Gata2^V/−^* HECs reached higher peak intensities (52.6 ± 2.7) than *Gata2^V/+^* HECs (45.8 ± 1.5, **, P = 0.0056; Fig. 6 E). BCs and IAHCs showed no peak MFI differences between *Gata2^V/−^* and *Gata2^V/+^* cells. The fold increase in trough-to-peak amplitude (Fig. 6 F) in *Gata2^V/−^* HECs did not change compared with *Gata2^V/+^* HECs. However, 11% of *Gata2^V/+^* BC showed trough-to-peak amplitudes higher than twofold, whereas only 4% of *Gata2^V/−^* BCs showed values above twofold. Among the few *Gata2^V/−^* IAHCs with at least one peak, the trough-to-peak amplitudes were similar to the values observed in *Gata2^V/+^* IAHCs. Together, our results show that Venus expression levels and pulsatile characteristics are altered during EHT in *Gata2* heterozygous mutant embryos as compared with embryos with normal levels of *Gata2* expression.

## Discussion

We have uncovered a new level of dynamic regulation involving the pulsatile expression of the pivotal *Gata2* TF during the establishment of hematopoietic cell fate in the embryo. Although genetic experiments implicate a role for *Gata2* in EHT cell populations, vital imaging of *G2V* EHT cells reveals for the first time pulsatile expression at the single cell level. Pulse parameters, as characterized by amplitude and periodicity of Venus expression in individual cells differs between the EHT subsets (Fig. 6 G). The HEC to BC transition is accompanied by an increase in reporter expression levels and increased pulsatile behavior. Expression further increases and stabilizes during the transition to IAHCs, with the periodicity and amplitude decreasing. Our results suggest that the high degree of pulsatile Gata2 expression in BCs is linked to cell fate transition during EHT and may reflect an active process involving the partial assembly of counterbalancing regulatory states (Kueh et al., 2016). This is supported by pulsatile and level changes in Gata2 expression that accompany a Gata2 heterozygous mutant state in which EHT is disrupted.

### Imaging dynamic cell transitions and Gata2 expression during EHT

Importantly, we used a *G2V* mouse model that does not disrupt *Gata2* expression levels or the function of the Gata2 protein (Kaimakis et al., 2016). The recombination of an *IRES-Venus* fragment into the 3′ UTR avoids hypomorphic *Gata2* expression and protein dysfunction that may result from a fusion protein. We showed previously that mice with two *G2V* alleles are normal in terms of HSC numbers and function. Gata2 protein has a relatively short half-life of 30–60 min (Minegishi et al., 2005; Lurie et al., 2008) as compared with other hematopoietic TFs such as Runx1 (3.3 h; Lorsbach et al., 2004) and Gata1 (4 to >6 h; Minegishi et al., 2005; Lurie et al., 2008). Its instability is related to ubiquitination (Minegishi et al., 2005; Lurie et al., 2008). The Venus reporter used in the *G2V* model has a half-life of ∼120 min (Li et al., 1998) and provides an excellent reporter of promoter activity, as it has a very short fluorescent protein formation (folding) time as compared with GFP (Snapp, 2009).

Our imaging (Fig. 2) and FACS (Table 1) experiments showed more cells undergoing EHT in the *G2V* embryonic aorta than previously described for the *Ly6aGFP* model (Boisset et al., 2010; Solaimani Kartalaei et al., 2015). At E10, Gata2 is expressed in ∼1,076 aortic ECs (CD31^+^ckit^−^) and ∼680 IAHC (CD31^+^ckit^+^), whereas Ly6aGFP is expressed in two to seven times fewer aortic ECs (190–831) and IAHCs (97–261). This is at the time when IAHCs peak and indicates that Gata2 expression marks more ECs with hemogenic potential and most if not all IAHCs. This is supported by functional data in which ∼80% of E11 AGM CFU-C are V^+^ (Fig. 5 A), whereas only 33% are GFP^+^ (Solaimani Kartalaei et al., 2015). However, all AGM HSCs are V^+^ and GFP^+^ (Solaimani Kartalaei et al., 2015). Thus, Ly6aGFP is a developmentally later marker, and Ly6aGFP-expressing cells are likely to represent a subset of Gata2-expressing cells that will have greater multilineage hematopoietic (including lymphoid) and HSC potential.

### Levels of Gata2

The exact relationship between Gata2 levels and cell fate decisions remains unclear. Previous work demonstrated that *Gata2* dosage is important in regulating the quantity and functional quality of HSCs (Ling et al., 2004; Rodrigues et al., 2005; Tipping et al., 2009). It has been shown that *Gata2* expression is down-regulated during lineage commitment (Orlic et al., 1995), suggesting a role for Gata2 in early hematopoietic progenitors and HSCs. More recently it has been shown that Gata2 lies at the core of a network of genes involved in lineage specification and mixed lineage states (Olsson et al., 2016). Transcriptome analysis of CD150^high^ adult bone marrow HSCs (Guo et al., 2013) showed concurrent high levels of *Gata2* expression and Gata2 occupancy of megakaryocyte-erythroid lineage-related genes. These high expressing HSC showed a bias to the formation of more megakaryocyte-erythroid colonies.

We have shown previously in our *G2V* reporter mouse model that the Venus high-expressing fraction in the embryonic aorta contains differentiated basophilic cell types, myeloid cells and innate immune cells (Kaimakis et al., 2016). Here we confirm that HPCs can be found in the Venus high-expressing fraction and further show that all HSCs and a large number of HPCs are found in the Venus intermediate-expressing cell fraction. Thus, commitment to the myeloid lineage in the early embryo seems to be accompanied by increased levels of *Gata2*. In line with this, low-level overexpression studies in bone marrow HSCs using a tamoxifen-inducible *Gata2 ERT* construct (Tipping et al., 2009), mimicking physiological levels of *Gata2* expression, promoted self-renewal and proliferation of myeloid progenitors. Physiological higher levels of *Gata2* block lymphoid differentiation (Tipping et al., 2009) and negatively correlate with the occupation of Gata2 with lymphoid-related genes (Guo et al., 2013). Although these data suggest that Gata2 levels regulate commitment to specific hematopoietic lineages in the adult bone marrow, during development, when *Gata2* expression is initiated and its expression is increasing in EHT cell populations, its levels are unstable in individual cells. Thus, we propose that *Gata2* pulsatile expression (in combination with the onset/asynchronous expression and stability of other pivotal TFs such as Runx1; Kueh et al., 2016) is likely to play a role in the stochastic commitment to the hematopoietic lineage.

### Regulation of pulsatile expression behavior

At the transcriptional level, pathways such as Notch, β-catenin/Wnt, and fibroblast growth factor (Dequéant et al., 2006), form negative feedback loops with appropriate delay time for a pulsatile element to be translated and act at the starting point. The combination of negative and positive feedback loops prevents transcription from reaching a homeostatic steady state and maintains pulsatile expression (Purvis and Lahav, 2013). Transcription of *Gata2* in the AGM is positively regulated by Notch1, which is required for EHT and HSC development (Robert-Moreno et al., 2005; Gama-Norton et al., 2015; Souilhol et al., 2016). Gata2 is autoregulatory and maintains its own transcription (Grass et al., 2003; Burch, 2005; Kobayashi-Osaki et al., 2005; Martowicz et al., 2005). Also, Notch1 activates *Hes1*, and Hes1 represses *Gata2* expression specifically in AGM hematopoietic cells (Guiu et al., 2013). In the absence of Hes1, Gata2 expression is high (Guiu et al., 2013) and the number of cells in intra-aortic hematopoietic clusters is increased. However, persistent high-level Gata2 expression results in nonfunctional HSCs (Tipping et al., 2009).

The positive and negative signals induced by the Notch pathway result in a so-called type I incoherent feed-forward loop (Mangan and Alon, 2003). In the case of *Gata2* in the embryonic aorta, we predict that the Notch–Hes1–Gata2 feed forward loop is responsible for the pulsatile expression of G2V that we observed in the EHT cell subsets: Notch1 would stimulate *Gata2* and *Hes1* transcription, and *Gata2* transcription would be repressed when Hes1 protein reaches a critical threshold (half-life, 24 min; Yoshiura et al., 2007), resulting in a pulse-like dynamics of Gata2 protein levels when Hes levels subsequently drop. That Hes1 is the likely pacemaker of *Gata2* pulsatile expression is supported by our RNA-sequencing data showing that *Hes1* is fourfold and *Notch1* sixfold up-regulated in the CD31^+^ckit^+^Venus^med^ fraction, as compared with the CD31^+^ckit^+^Venus^high^ fraction. Moreover, the higher expression of *Gata2* compared with *Hes1* in the CD31^+^ckit^+^Venus^high^ fraction suggests that critical Notch signaling thresholds will impact *Gata2* expression parameters in BCs versus IAHCs.

The in vivo G2V reporter allows for the first time the unbiased real-time characterization of *Gata2* expression during EHT in the *Gata2* WT and heterozygous mutant state. The mutant Gata2 mouse model has a deletion of the second zinc-finger domain in the *Gata2* gene (Tsai et al., 1994), leaving *Gata2*-binding motifs available on both alleles. Therefore, the altered *Gata2* pulsatile expression behavior in heterozygous mutant ECs and BCs cannot be explained by more Notch1 and Hes1 binding to only one allele of *Gata2*. Only half of the dose of DNA-binding Gata2 protein would be available to bind two alleles of *Gata2*, strongly suggesting that a reduced positive autoregulation manifests itself in altered Gata2 dynamic expression. As yet, we do not have a direct correlation between the levels of Venus protein and Gata2 protein in single cells of the AGM. In the future, mass spectrometry CyTOF (Giesen et al., 2014) could be used to more specifically address this issue. Further in vivo vital molecular studies, coupled with computational modeling of the interplay of *Gata2* regulators will be needed for a detailed understanding of the molecular basis of pulsatile expression. Given the fact that in hematopoietic disorders and malignancies *GATA2* mutations occur in the second zinc finger (Bresnick et al., 2012), the dysregulation of Gata2 pulsatile expression through a feed-forward loop might provide a mechanistic basis for human hematologic pathophysiologies.

## Materials and methods

### Mice and embryo generation

*Gata2^V/+^* conceptuses were generated by crossing *G2^V/V^* males (Kaimakis et al., 2016) with C57BL/6J females, and *Gata2^V/−^* conceptuses were generated by crossing *G2^V/V^* males with *Gata2^+/−^* females (five or more generations backcrossed onto C57BL/6J). *G2V* (*G2^V/V^*) and *Ly6aGFP* (*Ly6a^GFP/+^*) mice were mated to obtain E10.5 *G2V:Ly6aGFP* embryos (de Bruijn et al., 2002; Boisset et al., 2011). Quick genotyping (Kapa) of *Gata2^V/−^* embryos was performed by PCR using the following primers: *mGata2 39* (5′-GGAACGCCAACGGGGAC-3′), *mGata2 208* (5′-GCTGGACATCTTCCGATTCCGGGT-3′), and *Neo 503* (5′-GATCTCCTGTCATCTCACCTTGCT-3′; Tsai et al., 1994). Day of plug discovery was considered as E0 of embryonic development. Embryos were staged by SPs as E10 (28–40 SPs), early E10 (28–34 SPs), E10.5 (35–40 SPs), and E11.5 (40–50 SPs). All experiments were conducted according to Dutch and UK law and approved by the Dutch animal experiment committee (Stichting DEC consult) and the UK Animals Scientific Procedures Act 1986 Project License 70/8076, respectively.

### Embryonic tissue isolation and cell preparation and flow cytometry

Single cell suspensions of dissected AGM tissues (including part of the vitelline and umbilical arteries) were prepared by 45-min collagenase treatment (type I; Sigma) at 37°C and subsequently dissociated by filtering. For flow cytometric analysis, cells were incubated (30 min, 4°C) in PBS + 10% FCS + 1% PS with directly conjugated antibodies against CD31, CD45, and ckit. Embryos were, if necessary, genotyped (quick genotyping kit Kapa) and analyzed separately. Stained cells were analyzed or sorted on a SORP-FACSAria II flow cytometer (BD) equipped with a 488 blue laser and BB (“B,” blue laser; “B,” detector B) 454/35-bandpass filter to optimally detect Venus fluorescence. Dead cells were counter labeled with Hoechst and excluded from analysis and sorting.

### Whole-mount imaging

*G2V* whole-mount conceptuses were immunostained for CD31 and ckit as previously described (Yokomizo et al., 2012). Briefly, conceptuses were collected, fixed in 2% PFA and stored in methanol before labeling. Conceptuses were imaged in 50% benzyl alcohol/benzylbenzoate (1:2)/50% methanol using a laser scanning confocal microscope (SP5; Leica). Signals were collected sequentially to avoid bleed through. Three-dimensional images were reconstructed using Fiji imaging software.

### Time-lapse imaging and detection of Gata2 dynamics

Aortic transversal sections of E10 *G2V* embryos were prepared as previously described (Boisset et al., 2010). Briefly, nonfixed E10 (32–37 SP) embryos were freed from placenta, yolk sac, amnion, and head. Antibodies against CD31 and ckit (diluted in PBS/10% FCS/1% PS) were directly injected into the embryonic aorta. Transversal aortic slices of 150 µm width were cut with a tissue chopper (McIlwain). Draq5 (BioLegend) staining was performed on transversal G2V sections (15 min, RT, diluted in PBS/10% FCS/1% PS), after which sections were washed twice. Selected sections (from trunk to hindlimb) were subsequently embedded in 1% agarose in PBS and after polymerization overlaid with myeloid long-term culture medium (MyeloCult; StemCell Technologies) containing hydrocortisone and IL-3. Confocal time-lapse imaging was performed using a Leica SP5 microscope, equipped with 405-nm, argon, 561-nm, and 633-nm laser lines using a 20×, 0.7-NA air objective and typically a pinhole of 1–1.5 AU. Videos were recorded at a time interval of 15 min for a total of 12–15 h. For each experiment, three to five aorta slices, with a z-range of 20–50 steps (step size, 0.7–2.5 µm) were imaged. The sample temperature was maintained by a stage heater (37°C) and the sample was kept under constant CO_2_ levels (5%). The G2V signal was collected using an avalanche photo diode (APD) with a BP 535–585 emission filter, whereas the CD31-AF647 signal was collected with a photomultiplier tube (PMT) and a BP 650–720 emission filter. ckit-DyLight405 or ckit-BV421 were typically only imaged at the first frame of the time-lapse imaging series and detected with a BP 420–480 emission filter. To ensure that the Venus MFI was comparable between experiments, the microscope settings (laser power, gain, and settings of the emission filters) were kept similar among experiments.

### Image processing

To improve signal-to-noise ratios for more accurate tracking and object recognition, time-lapse imaging series were deconvolved using the Huygens Professional (Scientific Volume Imaging) Deconvolution Wizard. Small drifts in z and xy were corrected by the Huygens Professional Object Stabilizer. Deconvolved and stabilized time series were used for further analysis.

### Quantification of Gata2 dynamics

To analyze the dynamics of Venus expression in single cells in the aorta, Venus^+^ cells had to be tracked and the Venus fluorescence signal corresponding to individual single cells had to be extracted. Because no commercial tool was available that reliably tracked Venus expressing cells in the aorta and at the same time extracted the fluorescent signal, we developed a custom-made code to combine two commercial tools (1) tracking Venus^+^ cells (Huygens Professional Object Tracker) and (2) extracting voxel information (Huygens Professional Object Analyzer) of the Venus fluorescent signals in three dimensions and in time. Object Tracker and Object Analyzer use different algorithms to track and segment objects; therefore each tool assigned a unique identifier to each object (cell). Because both tools use the center of mass to describe the position of the object, our LabVIEW-based custom-made code assigned to each tracked object the closest segmented object with voxel information (within a maximum range of 5 µm). The resulting Venus^+^ cells with common identifier could be visualized in each time-lapse series by a custom-written Fiji macro. The tracked cells were visually inspected, and incorrectly tracked cells were excluded from further analysis. Moreover, further analysis was limited to cells that could be tracked over at least 10 consecutive frames and did not show any bleaching or overall intensity changes caused by the microscope setup.

To quantify the dynamics of Venus expression, the LabVIEW code also computed the volumetric MFI values of each tracked cell, which was defined as the sum of all intensities divided by the number of voxels representing the cell (Fig. S2). For further analysis, MATLAB codes (version 2015b) were developed to plot the Venus MFI as a function of time (Fig. S2). As a control, the MFI values were plotted against the voxel values, confirming that quantitative changes in the fluorescent intensity were not due to tracking errors.

### Data analysis

To assess whether the Venus signal in the time series data undergoes quantitative changes, we adapted a public-domain MATLAB code (http://www.billauer.co.il/peakdet.html) to automatically detect significant extrema in our Venus MFI time series data (Todd and Andrews, 1999). To discriminate against “noise” (such as fluctuations introduced by imperfections of the image stack segmentation), the code only considered local maximum as significant if it differed from its neighboring minima by more than a predefined threshold (specified as a percentage of the mean intensity of the track). For the analysis in this article, we used a threshold of 15%. Visual inspection of the minima and maxima confirmed that ∼90% of peaks were correctly detected using this threshold. Tracks with incorrectly recognized peaks were excluded from further analysis. Additional codes calculated the number of peaks, oscillation periodicity, peak minimum and peak maximum, and trough-to-peak amplitude. The data were exported from MATLAB to excel for further analysis and plotted in GraphPad Prism 5.

### Hematopoietic assays

The methylcellulose colony-forming assay was performed as previously described (Medvinsky et al., 2008). CD31^+^ckit^+^V^med^ and CD31^+^ckit^+^V^high^ sorted E10 AGMs (including part of the vitelline and umbilical arteries) were seeded in triplicate in methylcellulose (1 ml per dish; M3434; Stem Cell Technology) with 1% PS and incubated for 10 to 12 d at 37°C, 5% CO_2_. Colonies were counted with a bright-field microscope. Transplantation experiments were performed as previously described (Medvinsky et al., 2008). Sorted CD31^+^ckit^+^V^−^, CD31^+^ckit^−^V^+^, CD31^+^ckit^+^V^med^, and CD31^+^ckit^+^V^high^ (Ly5.2/Ly5.2) cells of five to seven E11 AGMs were transplanted into 9.5-Gy irradiated (Ly5.1/Ly5.1) recipients together with 2 × 10^5^ spleen cells from the recipient strain. Peripheral blood was analyzed by flow cytometry for donor contribution by anti-Ly5.1/anti-Ly5.2 labeling 1 and 4 mo after transplantation. Transplanted recipients were scored as positive if the peripheral blood donor chimerism was ≥10%. Multilineage organ chimerism analysis (lymphoid and myeloid) was performed 4 mo after transplantation.

### RNA isolation mRNA-sequencing analysis

CD31^+^ckit^+^V^med^ and CD31^+^ckit^+^V^high^ E10.5 AGM cells of *G2^V/+^* embryos were sorted into PBS/50% FCS/1% PS. After centrifugation and removal of supernatant, cells were lysed, and RNA was isolated using the mirVana miRNA Isolation kit (Ambion) according to the manufacturer’s protocol. RNA quality and quantity were accessed by the 2100 Bioanalyzer (Picochip; Agilent Technologies). RNA samples were prepared by SMARTer protocol. Illumina TrueSeq v2 protocol was used on HiSeq2500 with a single-read 50-bp and 9-bp index. Reads were aligned to the mouse genome (GRCm38/mm10) using Tophat/Bowtie, and the generated count table was analyzed by R/Bioconductor package edgeR according to McCarthy et al. (2012). Counts were normalized for mRNA abundance, and differential expression analysis was performed using edgeR. The B-H method was used for p-value correction with a false discovery rate of 0.05 as statistically significant. Variance stabilized counts were calculated by R/Bioconductor package DESeq for all genes (Anders and Huber, 2010). Cufflinks was used to compute transcript abundance estimates in fragments per kilobase per million (FPKM; Trapnell et al., 2013). For differentially expressed genes, the FPKM for each gene across all samples were normalized by division with maximum FPKM observed for that gene. Differentially expressed genes were analyzed for the top five most enriched Ingenuity Pathway Analysis pathways against a background of all mouse genes by right tailed Fisher exact tests in a core analysis calculating the likelihood that this is due to random chance. The accession number for the RNA-sequencing data is Gene Expression Omnibus: GSE106072.

### SDS-PAGE and Western blot

4 × 10^4^ E10 Venus^med^ and Venus^high^ AGM cells were sorted from E10.5 *G2^V/+^* and *G2^V/−^* embryos (littermates), washed and centrifuged, and directly lysed in Laemmli sample buffer. 4.5 to 6.2 × 10^4^ *G2^V/V^* bone marrow mononuclear cells were sorted into Venus^−^, Venus^med^, and Venus^high^ cell fractions, subsequently lysed in RIPA buffer plus protease and phosphatase inhibitor, and sonicated. Then Laemmli buffer was added. Proteins were separated by SDS-PAGE and transferred to PVDF membranes (Millipore). Subsequently, proteins were detected by anti-Gata2, anti-Venus, anti-GAPDH, anti–β-actin, anti-Hsp90, and anti-Cohesin immunoblotting. After labeling, Western blots were scanned using the Odyssey imager (LI-COR Biosciences).

### Antibodies

For flow cytometry, cells were stained with anti–CD31-PE-Cy7 (clone 390; BD), anti–cKit-BV421 (clone 2B8; BD), anti–Ly5.1-APC (clone A20, BD), and anti-Ly5.2-Fitc (clone 104, BD) monoclonal antibodies. For microscopy, CD31-AF647 (clone 390; eBioscience) and ckit-DyLight405 (clone 2B8; eBioscience; conjugated by the authors to DyLight 405; Pierce) were injected. In Fig. S1 A, Gata2, Venus, β-actin, GAPDH, and heat-shock protein 90 were detected by mouse monoclonal anti-Gata2 (clone CG2-96; Santa Cruz), rabbit polyclonal anti-Venus (MBL), mouse monoclonal anti–β-actin, rabbit polyclonal anti-GAPDH (gift of C. Hansen; Santa Cruz), and mouse monoclonal anti-Hsp90 (gift of C. Hansen; BD). In Fig. S5 A, Gata2 and Cohesin (subunit SMC3) were detected by rabbit anti-Gata2 (Santa Cruz) and rabbit anti-Cohesin (Abcam) polyclonal antibodies.

### Statistics

The data were compared in GraphPad Prism 5 using Mann–Whitney *U* tests, Student’s *t* tests, and one- or two-way ANOVA with Bonferroni post test, as indicated. Errors in the frequency of oscillation periodicity were estimated by bootstrapping (resampling residuals approach). Error bars represent two times the standard deviation originated from fitting procedures.

### Online supplemental material

Fig. S1 shows a Western blot of sorted Venus^med^ and Venus^high^ cells, demonstrating that Venus protein levels correctly reflect levels of Gata2, and confocal images show the differential expression pattern of Gata2 and Ly6a in *Ly6aGFP:G2V* thick aortic sections at E10.5. Fig. S2 illustrates the image acquisition and processing pipeline to analyze Venus expression in embryonic sections during confocal time-lapse imaging. Fig. S3 shows the visualization of Venus expression peaks throughout the imaging session. Fig. S4 shows two examples in which Venus expression IAHCs undergo mitosis during the imaging session. Fig. S5 shows Gata2 expression characteristics in *Gata2* heterozygous mutant embryos. Videos 1, 2, and 3 show examples of Venus-expressing cells undergoing EHT during G2V time-lapse imaging. 

## Supplementary Material

Supplemental Materials (PDF)

Video 1

Video 2

Video 3
