# Supplementary material for: In vivo single cell analysis reveals Gata2 dynamics in cells transitioning to hematopoietic fate
Source: J Exp Med. 2018 Jan 2;215(1):233–48. doi: 10.1084/jem.20170807 (PMC5748852; doi:10.1084/jem.20170807)
Supplement: Supplemental Materials (PDF) [file JEM_20170807_sm.pdf]

The Journal of Experimental Medicine

SUPPLEMENTAL MATERIAL

Eich et al., https://doi.org/10.1084/jem.20170807

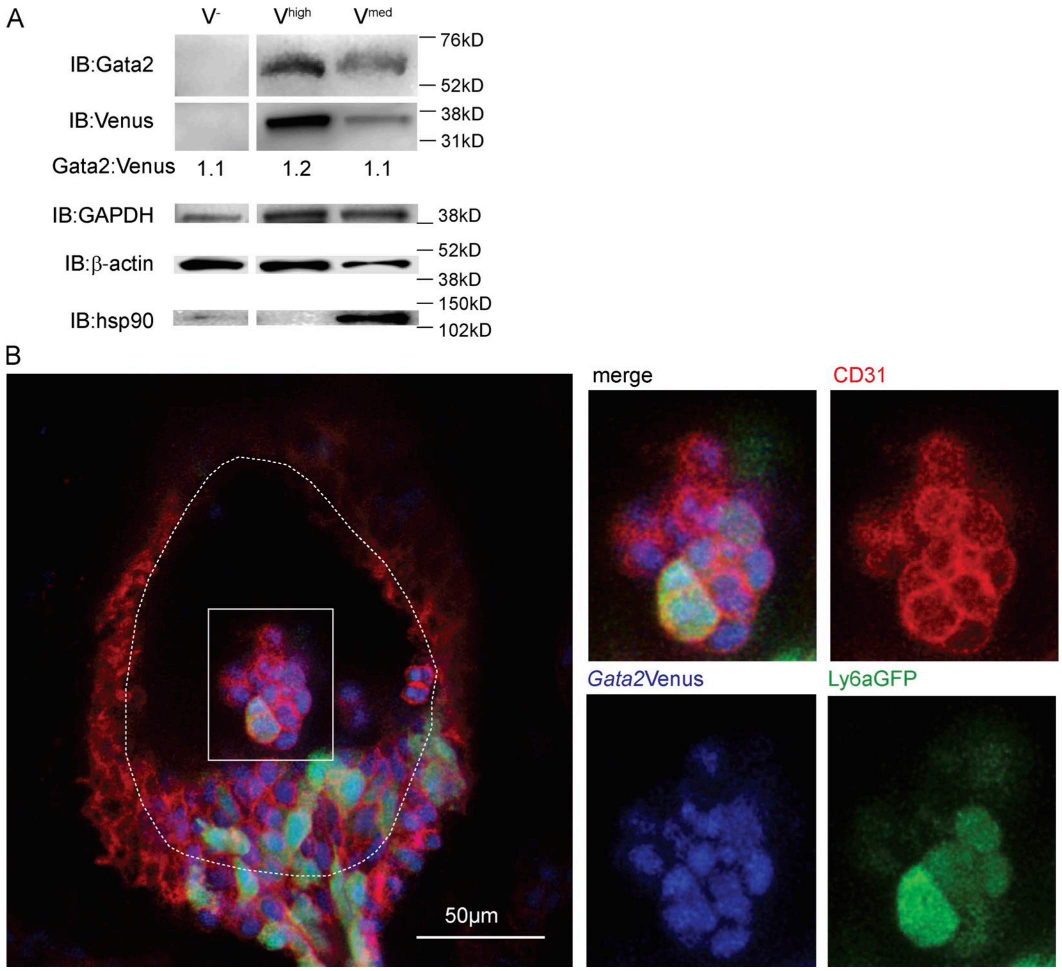

Figure S1. **Expression of Gata2, Venus, and Ly6aGFP. (A)** Representative Gata2 and Venus immunoblot (IB) of sorted Venus^{-}, Venus^{med}, and Venus^{high} cells of Gata2$^{V/V}$ bone marrow (*n* = 2). Although Venus^{high} and Venus^{med} sorted cells show Gata2 protein, Venus-negative sorted cells do not express Gata2 protein. Venus immunoblotting shows that the ratio of Gata2 protein to Venus protein is close to one in all cases, thus indicating a correlation in the protein levels of Gata2 and Venus. β-Actin, GAPDH, and Hsp90 immunoblottings were performed as protein loading controls but show high variability, suggesting that the sorted fractions contain hematopoietic cell types that differ in metabolism, cytoskeleton, etc. **(B)** Comparison of the *Ly6aGFP* and *G2V* reporter mouse models. Transverse section of *G2V:Ly6aGFP* E10.5 aorta. To compare Gata2 expression to the well-described stem cell marker Ly6aGFP during EHT, we simultaneously imaged both transgene reporters by crossing *G2V* (*G2V/V*) and *Ly6aGFP* (*Ly6aGFP/+*) mice (de Bruijn et al., 2002; Boisset et al., 2011) to obtain E10.5 *G2V:Ly6aGFP* embryos. Anti–CD31 antibody (red) was preinjected into the embryonic aorta (G2V, blue; Ly6aGFP, green). CD31-AF647, Ly6aGFP, and G2V were imaged sequentially. Albeit a similar expression pattern, cross sections of *Ly6aGFP:G2V* embryos at E10.5 showed a broader expression of G2V in EC and in IAHCs, as compared with Ly6aGFP. Although almost all IAHCs cells were Gata2 positive, only a few cells showed Ly6a expression. Bar, 50 µm. Related to Fig. 1 and Table 1.

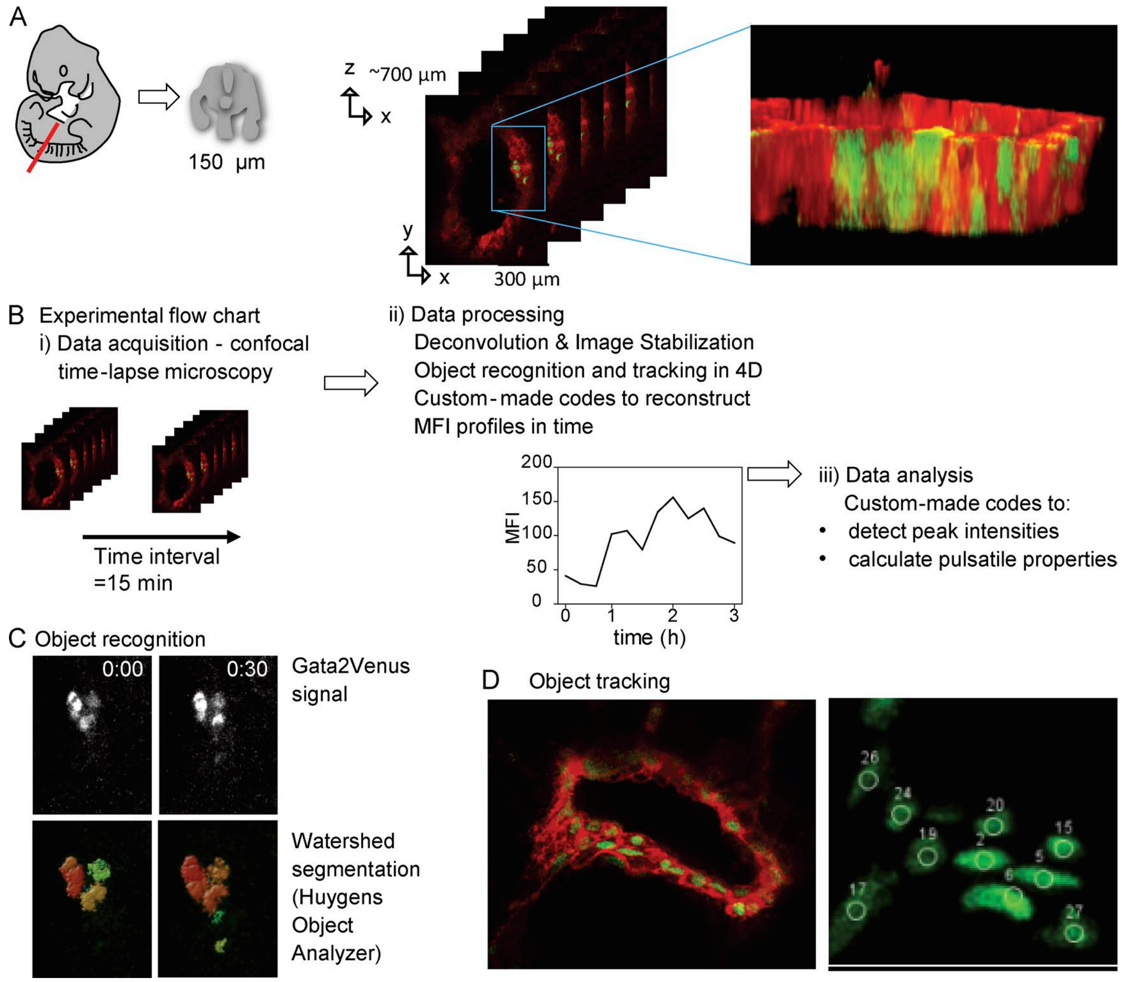

Figure S2. **Image acquisition and processing pipeline to display Venus MFI in four dimensions. (A)** Image acquisition. Images of *G2V* embryo slices were acquired at a spatial resolution of 300 nm in xy and 700–2,500 nm in z, at a time interval of 15 min, for up to 15 h. The mean width that was imaged per section was ~25–50 µm. **(B)** Image processing and analysis pipeline. Prior to analysis, images were deconvolved using Huygens Professional Deconvolution Wizard to improve the signal-to-noise ratio. To provide a direct view on Gata2 dynamics in single cells during EHT, Venus$^+$ cells were segmented in three dimensions using the Huygens Professional Object Analyzer and tracked in four dimensions using the Huygens Object Tracker. Custom-made codes combined both types of information to display the Venus MFI of single cells in four dimensions, as defined as the sum of all voxels/number of voxels per tracked object (see Materials and methods). MATLAB codes were developed to display the MFI as a function of time. Tracks of Venus$^+$ cells were excluded that showed bleaching or overall intensity changes caused by the microscope setup or cells present in < 10 consecutive frames. Additional MATLAB codes were developed to detect peaks in the Venus MFI data and compute the following parameters: peak number, peak intensity, oscillation periodicity, and trough-to-peak amplitude. **(C)** Example of object recognition using Huygens object analyzer on Venus deconvolved data. Single Venus$^+$ cells could be recognized in three dimensions using a watershed segmentation algorithm. **(D)** Visualization of Venus$^+$ cells recognized in four dimensions by our custom-made code. Our custom-made codes combined information of the object analyzer and object tracker and assigned new identifiers to the recognized cells. The result was visually inspected using a purpose-designed Fiji macro and corrected if necessary.

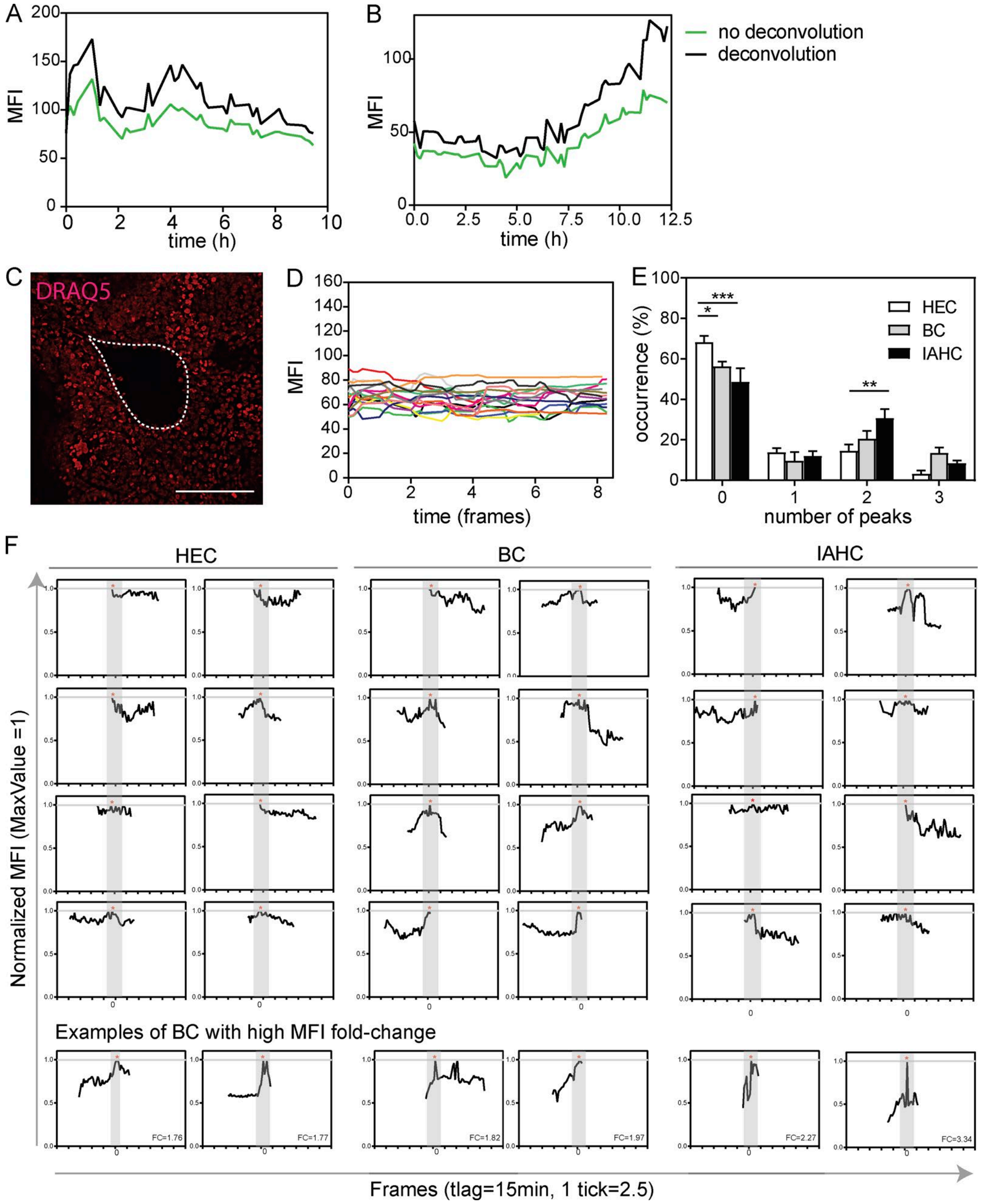

Figure S3. **Visualization of Venus expression peaks in confocal time-lapse imaging of aortic sections. (A and B)** Comparison of temporal variation of Venus MFI of individual Venus$^+$ cells in raw data versus deconvoluted data. A corresponds to Fig. 2 A and B to Fig. 3 B. **(C)** Confocal time-lapse imaging of E10 (33–36 SPs) transverse aortic section stained with DRAQ5 (red). Bar, 100 µm. Dashed line indicates the inner endothelial boundary. Transverse aortic sections were imaged for 10 h at a time interval of 15 min. **(D)** DRAQ5 MFI of individual cells plotted over time. **(E)** Distribution of pulse peak numbers in ECs, BCs, and IAHCs tracked over at least 10 consecutive frames (718 cells). The data represent the mean ± SEM (n = 15). Statistical significance was calculated using two-way ANOVA with Bonferroni post test (*, $P < 0.05$; **, $P < 0.01$; ***, $P < 0.001$). **(F)** Representative normalized Venus MFI tracks of each EHT subset (top) and BCs that undergo high fold change in MFI (bottom). Each track was normalized according to the local MFI maximum (=1, shown on the y axis and highlighted by a red asterisk). The local maxima of all tracks were aligned (shown in the gray bar). Images were acquired at a frame rate of 15 min, and each tick in the x axis represents 10 frames (2.5 h).

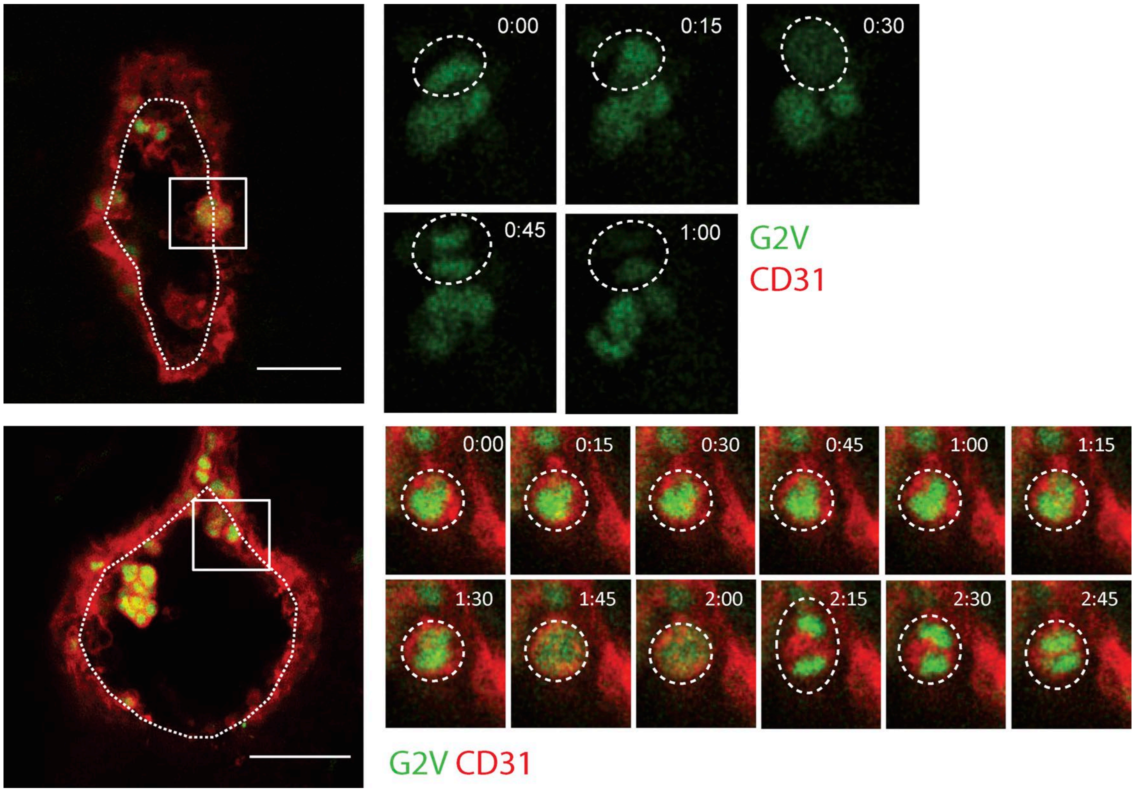

Figure S4. **Proliferation of Venus⁺ cells observed during time-lapse imaging.** Confocal time-lapse imaging of E10 (33–34 SPs) *G2V* embryos (Venus, green) stained with anti-CD31 (red) antibody. Transverse aortic sections were imaged for 10 h at time intervals of 15 min. Bars, 50 µm. Dashed circles indicate proliferation events observed in IAHCs.

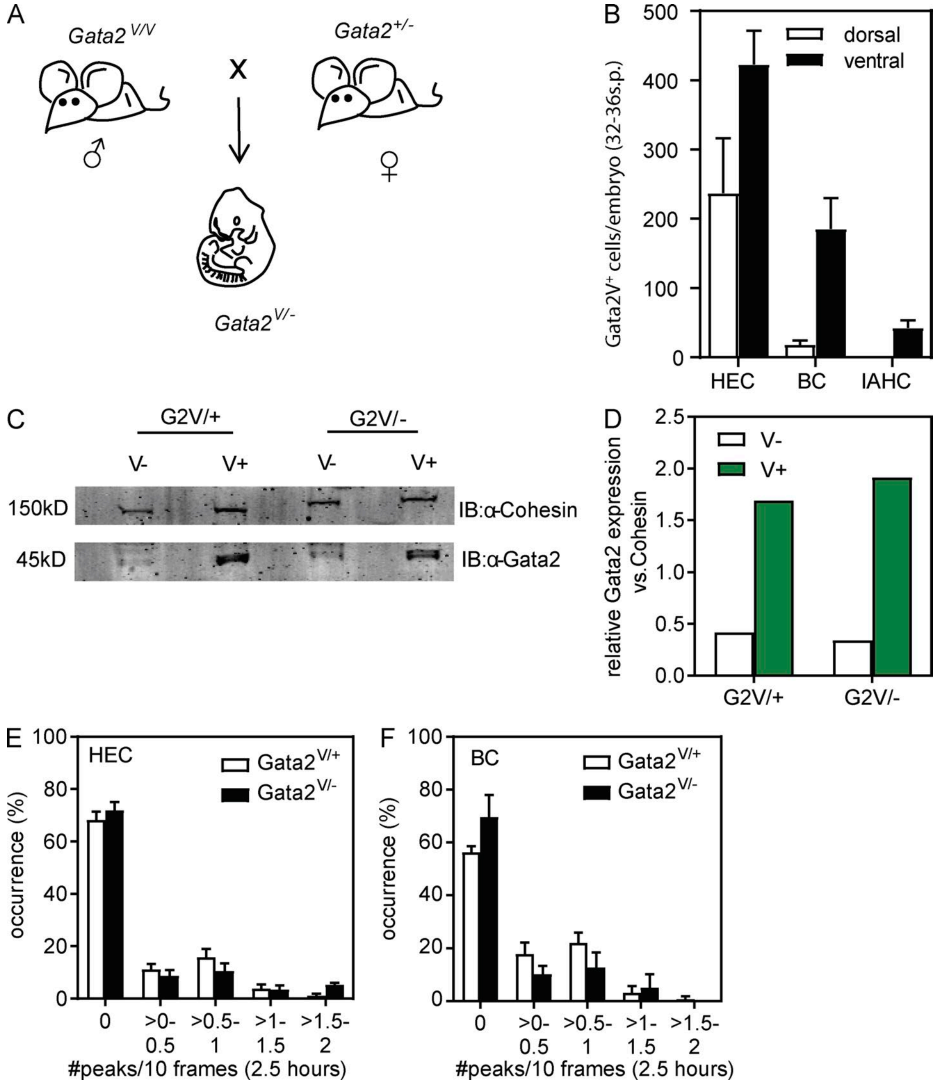

Figure S5. **Gata2 expression characteristics in *Gata2* heterozygous mutant embryos. (A)** Breeding scheme to obtain E10.5 *Gata2*$^{V/-}$ embryos: male G2V (*G2*$^{V/V}$) mice were crossed with *Gata2*$^{+/-}$ females. **(B)** Mean number of Venus^{+} EHT subset cells in *Gata2*$^{V/-}$ embryos per embryo (four to six independent experiments, 194 cells, and 12 slices). **(C)** Gata2 immunoblot of 4×10^4 sorted Venus^{+} cells of *Gata2*$^{V/+}$ and *Gata2*$^{V/-}$ E10.5 embryos. Cohesin labeling served as the protein loading control. **(D)** Quantification of Gata2 immunoblot signal relative to Cohesin signal. **(E and F)** Distribution of normalized Gata2 pulse peak numbers in *Gata2*$^{V/+}$ and *Gata2*$^{V/-}$ embryos in HECs (E) and BCs (F). Cells were tracked over at least 10 consecutive frames. To normalize for difference in track length, the data are presented as peaks per 10 frames (2.5 h) and represent the mean ± SEM (n = 15). Related to Fig. 6.

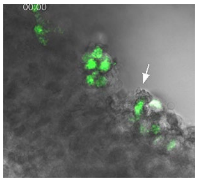

Video 1. **Confocal time-lapse imaging of E10 (33–34 SPs) *G2V* embryo thick section.** Transverse aortic sections were imaged for 10 h at 15-min intervals (Venus, green). Arrow indicates EHT events. Related to Fig. 2 A.

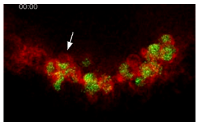

Video 2. **Confocal time-lapse imaging of E10 (33–34 SPs) *G2V* embryo thick section stained with anti–CD31 (red) antibody.** Transverse aortic sections were imaged for 10 h at 15 min intervals (Venus, green). Arrow indicates EHT events. Related to Fig. 2 B.

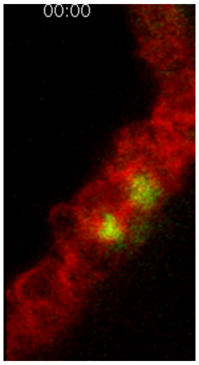

Video 3. **Confocal time-lapse imaging of E10 (33 SPs) *G2V* thick transverse aortic section stained with anti–CD31 antibody.** Transverse aortic sections were imaged for 10 h at a time interval of 15 min (Venus, green). Arrow indicates EHT events. Related to Fig. 2 C.

REFERENCES

Boisset, J.C., C. Andrieu-Soler, W.A. van Cappellen, T. Clapes, and C. Robin. 2011. Ex vivo time-lapse confocal imaging of the mouse embryo aorta. *Nat. Protoc.* 6:1792–1805. https://doi.org/10.1038/nprot.2011.401

de Bruijn, M.F., X. Ma, C. Robin, K. Ottersbach, M.J. Sanchez, and E. Dzierzak. 2002. Hematopoietic stem cells localize to the endothelial cell layer in the midgestation mouse aorta. *Immunity.* 16:673–683. https://doi.org/10.1016/S1074-7613(02)00313-8
